# Molecular Population Genetics of Inversion Breakpoint Regions in *Drosophila pseudoobscura*

**DOI:** 10.1534/g3.113.006122

**Published:** 2013-07-01

**Authors:** Andre G. Wallace, Don Detweiler, Stephen W. Schaeffer

**Affiliations:** Department of Biology, The Pennsylvania State University, University Park, Pennsylvania 16802-5301

**Keywords:** *Drosophila pseudoobscura*, chromosomal inversion, breakpoints, linkage disequilibrium

## Abstract

Paracentric inversions in populations can have a profound effect on the pattern and organization of nucleotide variability along a chromosome. Regions near inversion breakpoints are expected to have greater levels of differentiation because of reduced genetic exchange between different gene arrangements whereas central regions in the inverted segments are predicted to have lower levels of nucleotide differentiation due to greater levels of genetic flux among different karyotypes. We used the inversion polymorphism on the third chromosome of *Drosophila pseudoobscura* to test these predictions with an analysis of nucleotide diversity of 18 genetic markers near and away from inversion breakpoints. We tested hypotheses about how the presence of different chromosomal arrangements affects the pattern and organization of nucleotide variation. Overall, markers in the distal segment of the chromosome had greater levels of nucleotide heterozygosity than markers within the proximal segment of the chromosome. In addition, our results rejected the hypothesis that the breakpoints of derived inversions will have lower levels of nucleotide variability than breakpoints of ancestral inversions, even when strains with gene conversion events were removed. High levels of linkage disequilibrium were observed within all 11 breakpoint regions as well as between the ends of most proximal and distal breakpoints. The central region of the chromosome had the greatest levels of linkage disequilibrium compared with the proximal and distal regions because this is the region that experiences the highest level of recombination suppression. These data do not fully support the idea that genetic exchange is the sole force that influences genetic variation on inverted chromosomes.

Drosophila chromosomal inversions that give rise to different arrangements of genes have a long history in population genetics and evolutionary genetics research. From their initial discovery as factors that suppress recombination (Sturtevant 1917), inversion mutations that reordered genes in the genome became a powerful genetic marker to assay naturally occurring genetic variation. The ability to identify inversions with simple cytological analysis proved to be very important in these early studies (Painter 1934). Collections of Drosophila species from natural populations revealed a wealth of gene arrangement polymorphisms and provided the first genetic based data to infer phylogenetic relationships (Dobzhansky and Sturtevant 1938; Wasserman 1982; Sperlich and Pfriem 1986). The discovery of geographical gradients or clines of gene arrangement frequencies as well as seasonal cycling provided indirect evidence for selection acting on a genetic polymorphism. Wright and Dobzhansky (1946) used population cages of gene arrangement polymorphisms in *Drosophila pseudoobscura* to provide experimental evidence that selective effects could be replicated in the laboratory and that one could estimate the fitness differences among karyotypic genotypes. The ability of inverted chromosomes to suppress recombination (Sturtevant and Beadle 1936; Dobzhansky 1950; Kirkpatrick and Barton 2006; Kirkpatrick 2010) has suggested that this source of variation may play a role in adaptation by maintaining particular combinations of genes together.

Although chromosomal rearrangements have the potential to play a role in maintaining associations of alleles among multiple loci, inversion mutations can also shape nucleotide diversity within and outside of the inverted DNA segments in the absence of selection. Recombination plays an important role in homogenizing nucleotide variability between homologous chromosomes. New chromosomal inversion mutations are a potent isolating mechanism between homologous chromosomes that can lead to the differentiation of chromosomes with different gene orders. First, a newly inverted chromosome will capture a unique set of alleles based on the chromosome that was inverted initially. Second, new nucleotide mutations within arrangements are not likely to spread to different arrangements leading to differentiation of chromosomes. Recent theoretical studies have developed models to help understand how the presence of different gene arrangements can alter the pattern and organization of nucleotide variability along an inverted chromosome (Navarro *et al.* 1997, 2000). Navarro *et al.* (1997) examined how crossing over and gene conversion influence genetic exchange or flux among inverted chromosomes. Their analyses were performed using either a Poisson or a Counting model to determine the distribution of genetic exchange events along the chromosome. In the Poisson model, it is assumed that recombination events are independent while the Counting model assumes interference can occur among recombination events.

The reduction of recombination in gene arrangement heterozygotes results from the production of unbalanced gametes (Sturtevant and Beadle 1936; Roberts 1976). Not all crossing over events, however, produce unbalanced gametes either due to double crossovers or gene conversion. Gene-conversion events are cases in which small tracts of DNA, typically on the order of 300−400 nucleotides (Rozas and Aguadé 1990, 1993, 1994; Schaeffer and Anderson 2005), are exchanged between chromosomes. This nonreciprocal transfer of genetic information can prove to be more influential as a mechanism of genetic exchange among inverted chromosomes than reciprocal exchange in the form of crossing over (Navarro *et al.* 1997). The *rosy* locus found within inverted segments of the third chromosome of *Drosophila melanogaster* was shown to have a higher rate of exchange among heterokarytypes initiated by gene conversion rather than crossing over (Chovnick 1973; Hilliker *et al.* 1994). The Counting Model, which assumes crossovers interfere with one another, provided a better explanation for these observations by suggesting a one order of magnitude greater rate of recombination caused by gene conversion *vs.* crossing over within inverted regions (Navarro *et al.* 1997).

Navarro *et al.* (1997) showed that genetic exchange varies among the proximal, central, and distal regions of inverted chromosomes when a Counting model is assumed. Proximal regions (closer to the centromere) have lower levels of exchange than distal regions (nearer to the telomere). This predicts that proximal regions will diverge more among different gene arrangements than distal regions.

The size of an inversion can also determine how effective genetic exchange can be in differentiation among gene arrangements. Gene conversion rates are expected to be constant across inverted regions, but viable cross over products can only occur if two exchanges happen within the inverted segment. As inverted segments increase in size, there is a greater probability for crossovers to reduce genetic differentiation with the central regions being the most likely region to be exchanged among arrangements (Betran *et al.* 1997; Navarro *et al.* 1997). Gene conversion occurs at a constant rate across inversions of all sizes, but the short segment of DNA exchanged limits its power to reduce differentiation (Betran *et al.* 1997; Schaeffer and Anderson 2005). This leads to the general prediction that regions closest to breakpoints are likely to be more diverged than central regions of inversions because genetic exchange is lowest near the breakpoints (Navarro *et al.* 1997, 2000).

The reduction of recombination is also expected to lead to high levels of linkage disequilibrium (LD) or nonrandom associations among nucleotide sites. Because new mutations occur on a single chromosome, they generate nonrandom associations with variation on the chromosome. Recombination will shuffle variation on the chromosome breaking up the newly formed nonrandom associations. Because inversions prevent recombination from shuffling nucleotide diversity, one would predict that there should be high levels of LD between the proximal and distal breakpoints. LD is proposed as a method to map human genetic disease genes. The presence of inversions can lead to high levels of LD without being associated with disease genes. This study examines the structure of LD in the presence of paracentric inversions.

Navarro *et al.* (2000) explored the effects of chromosomal inversion polymorphisms on levels and patterns of nucleotide variability. Based on their models, they predicted that newer inversions will have lower nucleotide variability at inversion breakpoints than the central regions of the inverted segment while older inversions will have greater variability at the breakpoints than within the inverted segment.

*Drosophila* species are ideal model systems to examine the predictions of Navarro *et al.* (1997, 2000). The gene content is conserved among *Drosophila* species across the genus (Muller 1940), but the order of genes has been shuffled through the accumulation of fixed inversion mutations. As might be expected, populations of most *Drosophila* species are polymorphic for paracentric inversions providing the raw material for lineage specific gene orders (Sperlich and Pfriem 1986). For example, *D. pseudoobscura* populations have more than 30 different gene arrangements on its third chromosome that were created by a series of overlapping paracentric inversions (Dobzhansky and Epling 1944; Powell 1992). Inversions in *D. pseudoobscura* provide an excellent system to determine how mechanisms of genetic exchange alter the pattern and organization of nucleotide variability and divergence within and among the regions of *Drosophila* gene arrangements. Schaeffer *et al.* (2003) examined the pattern and organization of nucleotide diversity for eight genetic markers on the third chromosome. The data from the eight markers were used to reject part of the coadapted gene complex model for the maintenance of inversions originally proposed by Dobzhansky (1949). Only one of the eight marker loci (vestigial) mapped near an inversion breakpoint in the Schaeffer *et al.* (2003) study, while the other seven loci mapped well away from the breakpoints. To better test how genetic exchange on inverted chromosomes affects the pattern and organization of nucleotide diversity, we developed ten new genetic markers for the third chromosome that map near the proximal and distal inversion breakpoints of gene arrangements with high frequency and wide geographical distributions.

The availability of the *Drosophila pseudoobscura* genomic sequence data has enabled us to examine patterns and levels of nucleotide variability associated with regions in and around inverted chromosomes (Richards *et al.* 2005). In this study, strains of *D. pseudoobscura* from several different inversion types (chromosomal arrangements) were sequenced. The recent study of Wallace *et al.* (2011) has inferred the ancestral relationships among these gene arrangements. Regions near inversion breakpoints along the third chromosome of *D. pseudoobscura* were sequenced in ancestral and derived gene arrangements to test predictions about breakpoint evolution (Navarro *et al.* 1997; Navarro *et al.* 2000). First, estimates of nucleotide diversity were examined at each breakpoint region to see whether older inversions had higher levels of genetic variation at the breakpoints. Second, levels of variation in proximal regions, in the inverted regions, and in distal segments were estimated to test whether proximal regions are more divergent than distal regions as predicted by Navarro *et al.* (2000). Third, we derived two estimates of the neutral mutation parameter (4*N*_e_μ), π and θ, among all regions to test for departures from the neutral theory of molecular evolution (Kimura 1986). Finally, we estimated intralocus and interlocus LD to determine whether proximal regions have greater levels of LD than distal regions because of the polarity of genetic exchanges between these two segments of the chromosome and to determine whether nucleotide variation in the proximal and distal breakpoints is in significant LD.

## Materials and Methods

### Fly strains and DNA extraction

Strains of *D. pseudoobscura* were collected in 1998 from four different populations in the southwestern United States by S. W. Schaeffer and W. W. Anderson (University of Georgia). The localities include: Davis Mountain, TX; James Reserve, CA; Mount Saint Helena, CA; and Kaibab National Forest, AZ (Schaeffer *et al.* 2003). The third chromosomes from the strains collected from these localities were extracted using either the Blade or Lobe Balancer strain (Dobzhansky and Queal 1938). Genomic DNA was prepared from 141 isochromosomal lines of *Drosophila pseudoobscura* using a single-fly DNA extraction protocol (Gloor and Engels 1992).

### Polymerase chain reaction (PCR) primer design, amplification, and nucleotide sequencing

**Figure 1:**
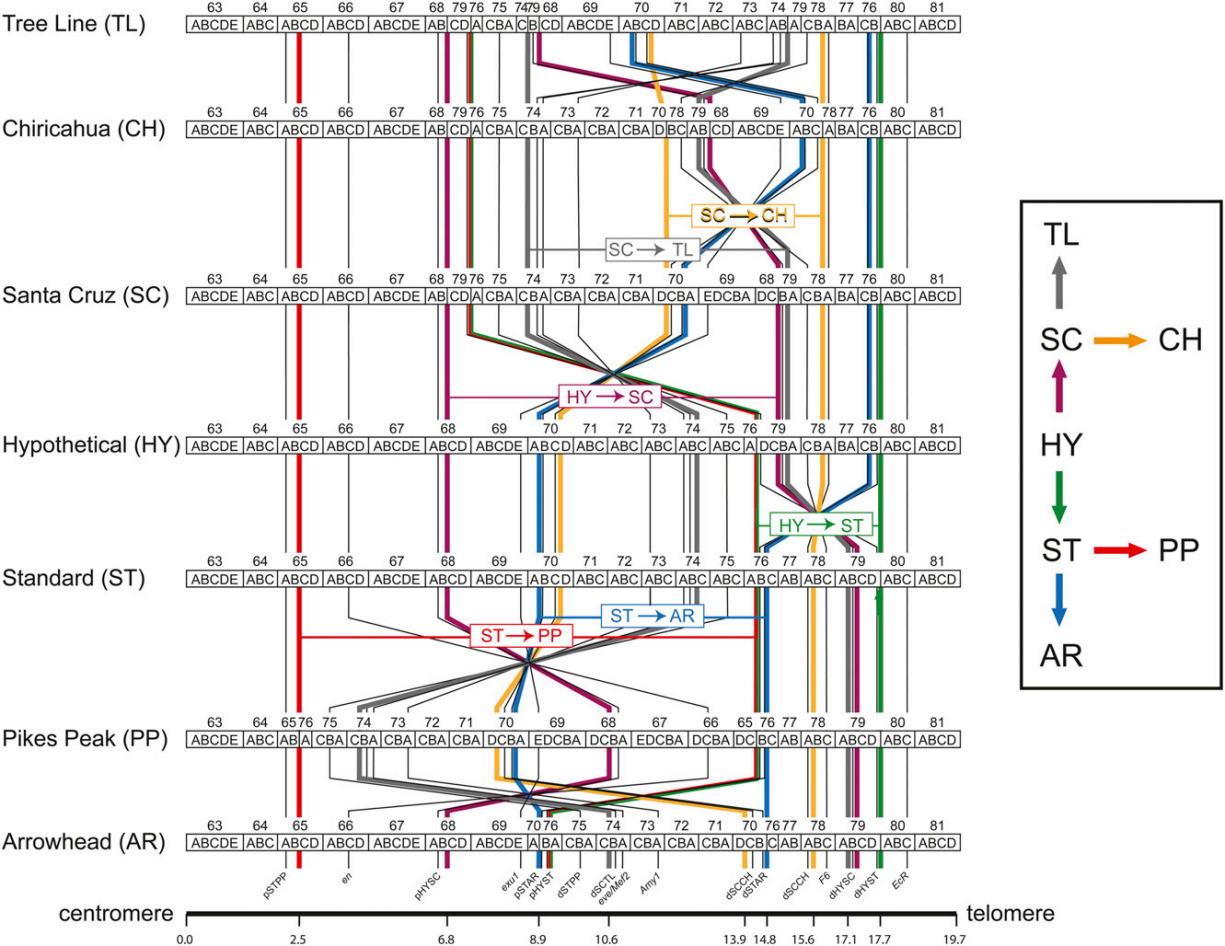
Map of 18 genetic markers on the gene arrangements of *Drosophila pseudoobscura*. The inversion events that gave rise the seven gene arrangements (TL, SC, CH, HY, ST, PP, and AR) are indicated by the colored arrows. The thick colored lines trace the locations of the six pairs of inversion breakpoints on the seven *D. pseudoobscura* gene arrangement cytogenetic maps, where the breakpoints are colored according the events in the right hand figure. For example, thick red lines represent the PP arrangement. The thin black lines show the locations of the 18 genetic markers that were sequenced in this study. The homologous marker and breakpoint positions on each gene arrangement are connected by vertical/diagonal lines.

A total of 18 gene regions were sequenced in this study. We used eight gene regions that were previously sequenced by Schaeffer *et al.* (2003)), engrailed (*en*), exuperantia 1 (*exu 1*), Myocyte enhancing factor 2 (*Mef 2*), even skipped (*eve*), amylase 1 (*Amy 1*), vestigial (*vg*), F6, and Ecdysone Receptor (*EcR*), and we used the primers and PCR conditions described in the study. These eight loci are evenly distributed across the *D. pseudoobscura* third chromosome. We designed PCR primers to amplify additional gene regions to be near the breakpoints of six polymorphic inversions using the third chromosome nucleotide sequence and cytogenetic map (Dobzhansky and Sturtevant 1938; Schaeffer *et al.* 2008). The gene arrangement breakpoints examined in this study are Pikes Peak (PP), Santa Cruz (SC), Tree Line (TL), Standard (ST), Hypothetical (HY) and Chiricahua (CH) (Dobzhansky 1944) (Figure 1). The vestigial locus (Schaeffer *et al.* 2003) was used as the marker for the distal ST to Arrowhead (AR) breakpoint because it is within 20 kb of the mapped AR distal breakpoint (Richards *et al.* 2005) requiring an additional 11 breakpoint markers to be developed. Because the SC and TL proximal breakpoint regions appear to be coincident on the cytological map (Dobzhansky 1944), only one primer pair was designed to interrogate sequence evolution at both inversion breakpoints. Thus, a total of 10 new markers were developed to examine the evolution of breakpoints for the six gene arrangements of *D. pseudoobscura* that are frequent and widely distributed in this species (Powell 1992). We used the comparison of the *D. persimilis* and *D. pseudoobscura* genome sequences of the third chromosome as a guide for choosing breakpoint regions that were likely to be less constrained at the nucleotide sequence level (Noor *et al.* 2007). We named the regions with a letter to signify the proximal (p) or distal (d) breakpoint, a two-letter code for the ancestral gene arrangement, and a two-letter code for the derived gene arrangement (Figure 1; Supporting Information, Table S1 with citation in File S2). The PCR fragments amplified for the breakpoint regions were between 375 and 1413 bp in length. Five of the sequenced breakpoint regions, pHYST, dSTPP, dSTAR, dSCCH, and dHYST, included some coding sequences. Total PCR volume was 50 µL which included 10 × polymerase buffer with MgCl_2_, both forward and reverse primers, deoxynucleotide triphosphate mix, Taq DNA polymerase, and the isolated target DNA. The 30-cycle PCR protocol included 2 minutes of initial denaturing at 95º, annealing at the temperatures specific to each primer pair (Table 1) for 30 sec/kb, and extension at 72º for 1 min/kb. The amplification product was verified as the predicted length by running 5 µL of the completed PCR on 2% agarose gel. Once the fragment size was verified, the completed PCR was treated with ExoSAP-IT (USB; Affymetrix Corporation) to remove contaminants such as unused deoxynucleotide triphosphates and primers remaining in the PCR mixture. Fragments with sequencing primer at a concentration of 1 μM were then sequenced at the Penn State University Nucleic Acid Facility (University Park, PA) using an ABI Hitachi 3730XL DNA Analyzer. Each breakpoint region was sequenced in both forward and reverse directions. The forward and reverse sequence reads were assembled and conflicts between the two reads were resolved using the SEQMANII program (DNASTAR, Madison, WI).

**Table 1:** Mean estimates of heterozygosity (π ± SD) within- and between-gene arrangements in proximal, inverted, and distal regions of the third chromosome of *D. pseudoobscura*

Type of variation	CH	AR	PP	ST	TL
Within arrangement (π)					
Proximal	0.010 ± 0.004	0.007 ± 0.004	0.011 ± 0.006	0.008 ± 0.004	0.006 ± 0.004
n	16	19	20	18	4
bp	3249	1031	355	1031	2043
Inverted	0.009 ± 0.004	0.009 ± 0.004	0.013 ± 0.006	0.007 ± 0.003	0.009 ± 0.005
bp	1475	3258	3293	3258	2254
Distal	0.014 ± 0.007	0.011 ± 0.005	0.017 ± 0.008	0.007 ± 0.003	0.013 ± 0.007
bp	1186	1620	1619	1619	1583
Among arrangements (π)	ST-AR	ST-PP			
Proximal	0.008 ± 0.004	0.011 ± 0.006			
n	37	38			
bp	1031	353			
Inverted	0.011 ± 0.005	0.015 ± 0.007			
bp	3258	2957			
Distal	0.010 ± 0.004	0.015 ± 0.007			
bp	1619	2597			

CH, Chiricahua; AR, Arrowhead; PP, Pikes Peak; ST, Standard; TL, Tree Line; n, sample size; bp, number base pairs in the region; π, estimate of heterozygosity based on the number of pairwise differences (Tajima 1983).

Seventy five strains had all 18 genetic markers sequenced, which enabled us to concatenate the data for LD analyses. Sixty-six strains were sequenced for at least one genetic marker. The sequences of 18 loci were extracted from Illumina next-generation sequencing reads for two additional strains, one inbred strain collected from Mather, CA that carried the TL arrangement and a second strain from the outgroup strain *D. miranda* SP138 (data kindly provided by M. A. F. Noor, Duke University). Thus, the number of strains sequenced for any of the 18 genetic markers varied from 86 to 144.

### Nucleotide sequence alignment

Finished nucleotide sequences for each of the 18 genetic markers were aligned using the MEGALIGN program (DNASTAR, Madison, WI). Alignments were performed manually and inspected to insure that indels were scored consistently among the different sequences.

### Nucleotide polymorphism and divergence at *D. pseudoobscura* breakpoint regions

We used the aligned sequences at the 18 markers to examine the predictions of the Navarro *et al.* (1997, 2000) models by estimating levels of genetic variation within each gene arrangement and among arrangements. DnaSP was used for these population genomic analyses (Librado and Rozas 2009). The input aligned sequence files were updated within DnaSP to identify coding regions in the alignment based on the annotated *D. pseudoobscura* genome (version R2.27). In addition, strains were placed into a variety of subsets based on single gene arrangements or combinations of two gene arrangements. All nexus format data files used in these analyses are available on the ScholarSphere web site (http://ScholarSphere.psu.edu). DnaSP was used to estimate nucleotide variability within all sequences at each locus. We tested the predictions of Navarro *et al.* (2000) by estimating how breakpoint position influences levels of nucleotide variability within and among five gene arrangements (AR, PP, CH, ST, and TL) with estimates of nucleotide heterozygosity based on the number of segregating sites (Θw) (Watterson 1975) and the number of pairwise differences (π) in silent sites (Tajima 1983). We estimated the variance of Θw based on a model of no recombination (Tajima 1983). Nucleotide heterozygosity was estimated within the derived and ancestral arrangements as well as across the two arrangements.

Estimates of nucleotide diversity used the Jukes and Cantor (1969) multiple hits correction. Wallace *et al.* (2011) has shown that the HY chromosome is the ancestral gene arrangement (Figure 1). Therefore, we polarized the inversion events on the *D. pseudoobscura* third chromosome phylogeny based on the HY chromosome being the common ancestor of all other arrangements. The estimated age (95% confidence limits) of the gene arrangements are: 2.01 (1.81−2.23) million years for HY, 1.38 (1.19−1.42) million years for SC and ST, 1.22 (1.09−1.34) million years for TL, 0.99 (0.88−1.10) million years for PP, 0.58 (0.51−0.65) million years for AR, and 0.51 (0.45−0.57) million years for CH. The confidence limits for the two oldest arrangements ST and TL overlap, but we assume that the TL is oldest extant arrangement based on migration-selection models (see page 3083 in Schaeffer 2008). The ages of the arrangements are critical for testing the models of Navarro *et al.* (1997, 2000) where levels of variation in ancestral and derived inversions are key features of the model. The relative ages of the inversions are also necessary for coalescent models (see *Tests of an equilibrium neutral model* below). Nucleotide variability (Θw and π) was estimated across the gene markers of the third chromosome in ancestral and derived arrangements to determine whether diversity was greater at the breakpoints compared to central regions (Navarro *et al.* 1997) and to determine whether derived inversions had lower levels of diversity than ancestral arrangements but increased in the sample of ancestral and derived arrangements (Navarro *et al.* 2000). Using Figure 1 as a guide, we ordered genes according to the derived arrangement in plots of heterozygosity along the chromosome.

### Tests of an equilibrium neutral model

The Tajima’s *D* statistic was used to test for departures from an equilibrium neutral theory of molecular evolution (Tajima 1989). We estimated values for Tajima’s *D* across all sequences at each locus and also within individual gene arrangements. In addition, the Hudson Kreitman, and Aguade (HKA) test (Hudson *et al.* 1987) was used to test for departures from a neutral model. We used the software written by Jody Hey (Rutgers University (http://genfaculty.rutgers.edu/hey/software), which allows a multiple locus test with varying sample sizes.

We used coalescent models with nested subsamples to test whether nucleotide diversity estimates within arrangements reject a neutral model (Hudson and Kaplan 1986; Schaeffer *et al.* 2003). Each genetic marker had different numbers of the six arrangements ranked from youngest to oldest (f_CH_, f_AR_, f_PP_, f_ST_, f_TL_, f_SC_), where f_CH_, f_AR_, f_PP_, f_ST_, f_TL_, and f_SC_ are the numbers of CH, AR, PP, ST, TL, and SC chromosomes, respectively. These coalescent simulations generated topologies and coalescent times for 5000 individuals using standard approaches (Hudson 1990); however, inversion and nucleotide mutations were generated on the branches of the tree rather than just nucleotide changes. First, inversion mutations were placed on the tree, then we counted the number of gene arrangements (*n*_i_) and their rank-ordered frequencies in the terminal descendants (f_1_, f_2_, f_3_, f_4_, f_5_, f_6_), where f_1_ is the least frequent and f_6_ is the most frequent. In a neutral genealogy, the frequency of alleles will positively relate with age (Toomajian *et al.* 2003). Thus, a genealogy was accepted if the tree had an *n*_i_ = 6 and if the rank-ordered frequencies were greater than or equal to the observed age-ordered frequencies (f_1_≥ f_CH_, f_2_≥ f_AR_, f_3_≥ f_PP_, f_4_≥ f_ST_, f_5_≥ f_TL_, f_6_≥ f_SC_). Nucleotide mutations were added to an accepted genealogy according to standard coalescent methods (Hudson 1990). Alleles were subsampled within each arrangement class up to the observed sample sizes of the different chromosomes (f_CH_, f_AR_, f_PP_, f_ST_, f_TL_, f_SC_). We estimated heterozygosity [θ_w_ (Watterson 1975); π (Tajima 1983)] within each arrangement and among all arrangements for each replicate genealogy. A total of 1000 random genealogies were sampled and a 95% confidence interval was inferred for the heterozygosity estimates within each arrangement and among all arrangements. These confidence limits were compared with the observed estimates of Θw and π to determine whether the observed values had more extreme values.

There is evidence that populations of *D. pseudoobscura* are exponentially growing at a modest rate (Schaeffer 2002). Thus, we used a coalescent model that incorporates exponential growth with rate parameter α = 7 (Slatkin and Hudson 1991). The methods for this coalescent were the same as above except that the coalescent times were determined for a model of exponential growth with rate parameter α = 7.

### LD analysis

Fisher’s exact test (Sokal and Rohlf 1981) was used to test pairs of variable sites within and between third chromosomal loci for significant LD. We concatenated the aligned sequences from the 11 breakpoint regions and seven gene regions of 76 strains to test for intra- and interlocus LD. Only comparisons were performed of sites capable of generating a significant result with the Fisher’s exact test (Lewontin 1995). We used the Q-value approach with a false-discovery rate of 1% to overcome the multiple comparison problem (Storey 2002). We estimated the fraction of valid tests of LD that showed significant nonrandom association within and between each region. We plotted these values as a heat map to show regions with low *vs.* high levels of LD. The numbers of significant associations within and between loci were tested for departures from homogeneity using a χ^2^ test. We also tested the nucleotide sites for significant associations with the five major gene arrangements. A separate Q analysis with a false-discovery rate of 1% was performed for the gene arrangement specific association tests. We estimated the fraction of valid tests of LD that were significant between each region and the gene arrangement type. We plotted these values as a heat map tested for the numbers of significant tests for departures from homogeneity using a χ^2^ test.

We also tested whether the LD increases as gene arrangements age. In the ST phylad, the PP arrangement is older than the AR arrangement. We asked whether an older arrangement accumulates more LD due to new mutations and reduced genetic exchange. We tested this hypothesis by creating two subsets of gene arrangement sequences. The first subset includes ST and AR arrangements representing LD for an ancestral and recent arrangement. The second subset includes ST and PP arrangements representing LD for an ancestral and an older arrangement. Each subset of data were tested for LD separately and the resulting probabilities from the two analyses were combined to determine an overall significance cutoff for the multiple tests using the Q-value approach and a common false discovery rate of 1%.

## Results and Discussion

### Tests of the impact of heterogeneous recombination rates on nucleotide polymorphism and divergence on inverted chromosomes in *D. pseudoobscura*

The history of the gene arrangements of the third chromosome of *D. pseudoobscura* reflect a complex history that is written in the observed pattern of nucleotide variation. The evolutionary history that is reflected in the sequences at breakpoints differs from that seen at non-breakpoint sequences. Navarro *et al.* (1997) used the Poisson and Counting models of recombination to predict levels of genetic flux across an inverted chromosome. The Poisson model assumes that crossing over and gene conversion events are independent, whereas the Counting model allows for interference of genetic exchange events. Their models showed that recombination rates will be lower in the proximal *vs.* distal chromosomal segment (Navarro *et al.* 1997), which predicts that proximal regions will have greater levels of nucleotide differentiation *vs.* distal segments of the inverted chromosome. A summary of all estimates of heterozygosity Θw and π in silent sites (synonymous sites in coding sequence plus noncoding sites) for the 18 genetic markers within and among all arrangements is shown in Table S2 with citations in File S2.

We examined this prediction by estimating heterozygosity in the proximal, inverted, and distal regions within the five gene arrangements based on the position of the 18 markers relative to the derived arrangement breakpoints (Figure 1). Proximal markers were between the centromere and the proximal breakpoint, inverted markers were between the two breakpoints, and the distal markers were between the distal breakpoint and the telomere. In addition, we examined heterozygosity among two pairs of gene arrangements to determine whether heterozygosity increases in these three regions as expected. Mean estimates of heterozygosity in the three regions within- and between-gene arrangements are shown in Table 1. Mean estimates of π are greater in distal regions than in proximal regions in all but the ST arrangement. The differences in heterozygosity, however, are not significantly different from each other, although four of the five arrangements show the same trend, despite different sequenced regions used in the estimates. The older arrangement (PP) shows greater levels of variation when pooled with its ancestral arrangement than does the younger arrangement (AR) as is expected. The mean nucleotide differentiation is greatest within the inverted regions and lowest in the proximal segments.

These data fail to support the theoretical predications of Navarro *et al.* (1997) with the proximal region showing less heterozygosity than the inverted or distal regions. This observation may suggest that proximal regions are subject to partial selective sweeps (Navarro *et al.* 1997, 2000). Regions that experience lower levels of recombination are expected to harbor less variation as a consequence of recurrent selective sweeps (Begun and Aquadro 1992). These results should be viewed with caution given the small number of loci sampled in each region. With the advent of next generation sequencing technologies it will now be possible to do more thorough examination of overall trends and increase the number of regions sampled across the chromosome.

### Selection removes and elevates variation on the *D. pseudoobscura* third chromosome

Geographic and altitudinal clines, seasonal cycling, and population cage experiments have been used as circumstantial evidence that selection acts on the gene arrangements of *Drosophila pseudoobscura* (Dobzhansky 1943; Wright and Dobzhansky 1946), but the nature of the selective regime has not been clear.

We tested the frequency spectra of each region within the five arrangements as well as all arrangements for departures from an equilibrium neutral model using Tajima’s *D* (1989) test statistic. Tajima’s *D* estimates the difference between two heterozygosity values, one based on the number of nucleotide differences between pairs of sequences and a second based on the number of segregating sites. The Tajima’s *D* will have significantly positive values when a region has an abundance of intermediate frequency variants perhaps indicating the action of some form of balancing selection. Alternatively, significant negative values indicate that a region has an excess of rare variants either because it is recovering from a selective sweep or may reflect a recent population expansion (Innan and Stephan 2000). One can discriminate between the selective and demographic hypotheses based on whether a few loci or a majority of loci in the genome have a negative *D*, respectively.

The results of the Tajima’s tests show a consistent trend toward negative values within and among gene arrangements with only six of the 108 potential tests showing a significant negative *D* (pSTPP in TL, pHYSC in ST, pSCCH in ST, dSTAR in CH, *en* in AR, *Mef2* in ST; Table S2). Two breakpoint regions showed a significant excess of intermediate frequency variants (*D* > 0) (pSCCH in PP, dSCCH in TL), whereas none of the nonbreakpoint regions had a *D* significantly greater than zero.

The overall trend of negative Tajima’s *D* values at a majority of loci in *D. pseudoobscura* supports a previous conclusion that the populations of *D. pseudoobscura* are expanding (Hamblin and Aquadro 1999; Schaeffer 2002; Schaeffer *et al.* 2003). There were a number of breakpoint regions that had a higher percentage of positive Tajima’s *D* values. These results suggest that long-term maintenance of multiple gene arrangements has raised the frequency of allelic variants near some breakpoints.

**Figure 2:**
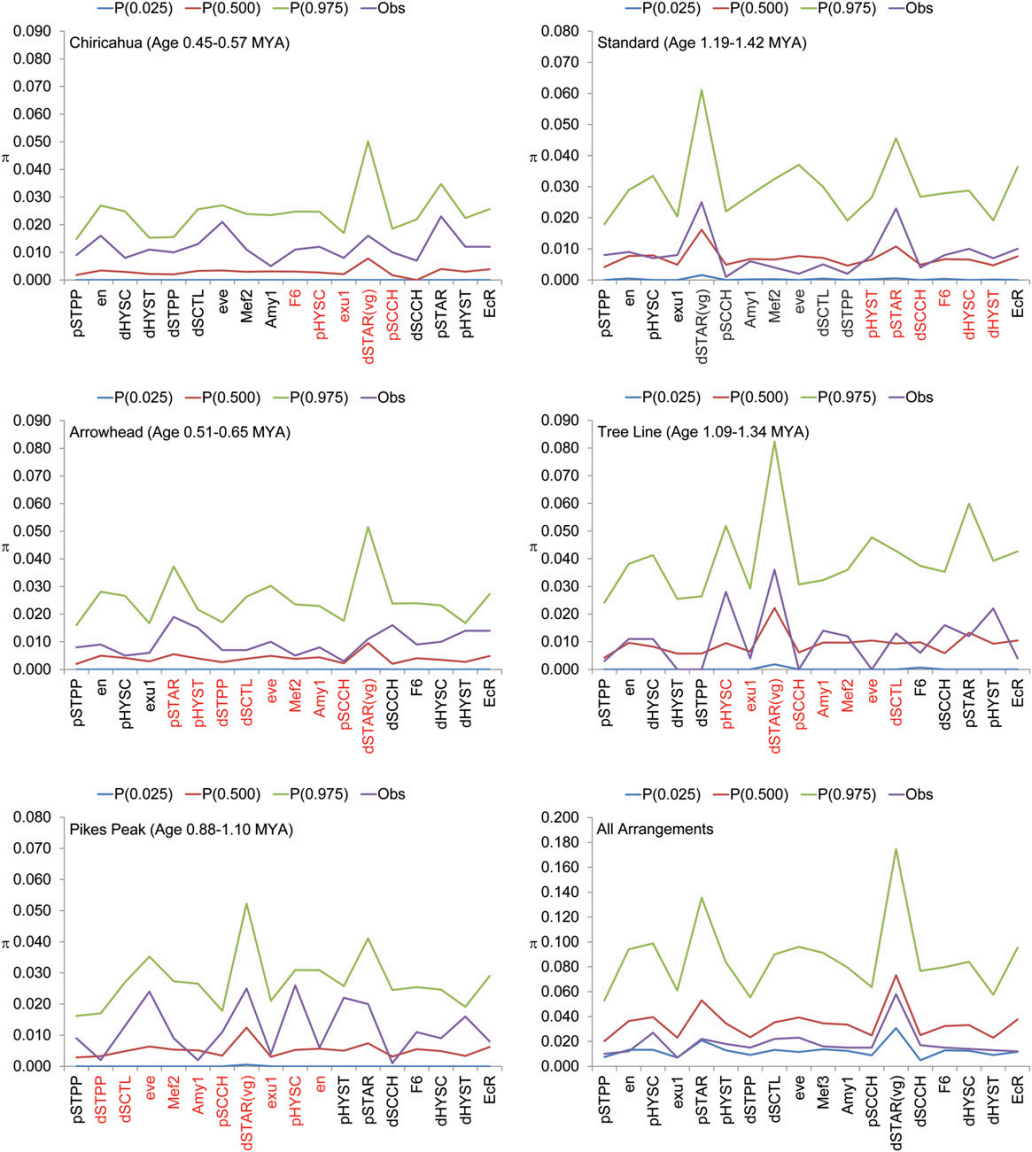
Observed *vs.* expected estimates of nucleotide heterozygosity (π) at 18 *D. pseudoobscura* marker loci based on a coalescent analysis of a population of constant size. Nucleotide heterozygosity estimates based on the number of pairwise differences (π) within five gene arrangements (AR, PP, CH, ST, and TL) as well as among all arrangements are shown in the individual panels. The observed (Obs) estimates of π as well as the mean (P(0.500) and the 95% confidence interval (P(0.025) to P(0.975)) derived from 1000 coalescent simulations using nested subsamples for a constant population size model. The genetic markers labeled in red on the x-axis are genes within the inverted region of the derived arrangement.

We used coalescent simulations with nested subsamples as a second approach to test whether levels of nucleotide variation within each arrangement depart from an equilibrium neutral model. These analyses ranked the arrangements according to their relative ages. Figure 2 shows that the estimates of heterozygosity (π, based on pairwise differences) across all arrangements are uniformly lower than the mean for the coalescent analysis assuming constant population sizes. In contrast, estimates of heterozygosity based on the number of segregating sites generally were greater for all loci and in some cases were greater than the 95% confidence interval (Figure S1). This result is expected given that the majority of loci in *D. pseudoobscura* have a negative Tajima’s *D*. All estimates of π for all loci within the five arrangements are within the 95% confidence limits derived from the coalescent analysis.

**Figure 3:**
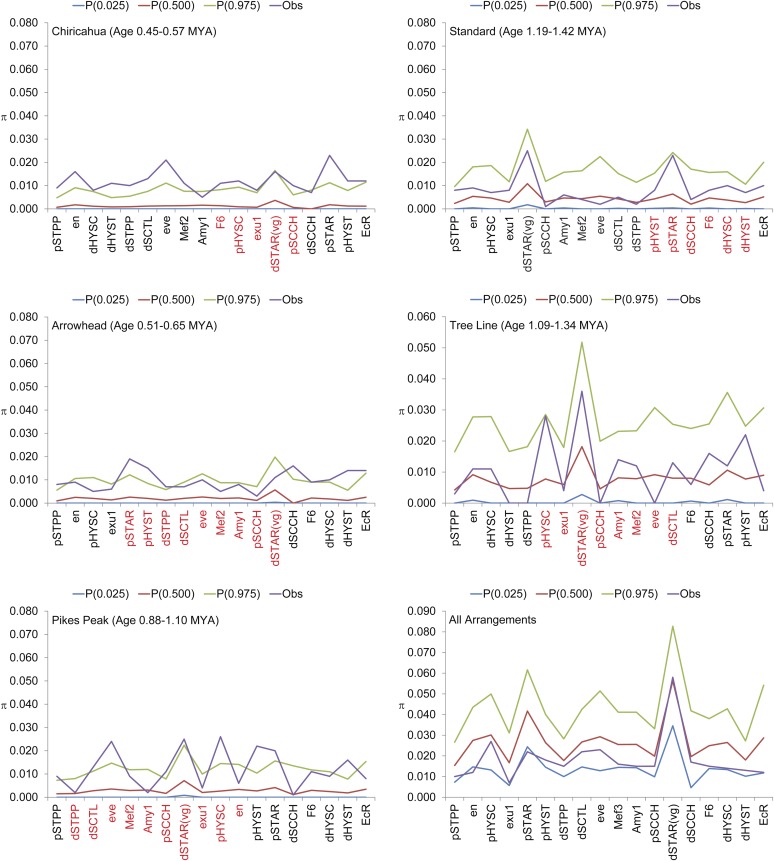
Observed *vs.* expected estimates of nucleotide heterozygosity (Θw) at 18 *D. pseudoobscura* marker loci based on a coalescent model of an exponentially growing population. Nucleotide heterozygosity estimates based on the number of segregating sites within five gene arrangements (AR, PP, CH, ST, and TL) as well as among all arrangements are shown in the individual panels. The observed (Obs) estimates of Θw as well as the mean (*P*(0.500) and the 95% confidence interval (P(0.025) to P(0.975)) derived from 1000 coalescent simulations using nested subsamples for an exponentially growing population with growth rate parameter α = 7. The genetic markers labeled in red on the x-axis are genes within the inverted region of the derived arrangement. The proximal region is to the left.

Figure 3 shows the results of coalescent simulations assuming an exponentially growing population with a growth rate parameter of α = 7 (Schaeffer 2002). The observed estimates of π across all arrangements matched the expected values (*P* = 0.5) for the 18 regions examined in the coalescent simulations assuming an exponentially growing population. The younger arrangements tended to show more observed heterozygosity (π) estimates outside of the upper confidence limit (*P* = 0.975) than older arrangements (Figure 3). Estimates of heterozygosity based on the number of segregating sites showed similar trends, but showed more loci with values outside the 95% confidence interval in younger arrangements (Figure S2)

Finally, we used the HKA test (Hudson *et al.* 1987) test to determine whether levels of polymorphism within the five arrangements are correlated with divergence between *D. pseudoobscura* and the outgroup *D. miranda*. We tested the 18 gene regions for deficiencies or excesses of variation. Only the TL chromosome of the five gene arrangements rejected an equilibrium neutral model with the HKA test (χ^2^ = 20.91, *df* = 17, *P* = 0.047; Table 2, Table S3, Table S4, Table S5, and Table S6 for the nonsignificant results for the other arrangements). The main reason for the rejection is the lack of polymorphic sites in the dHYST region despite high levels of divergence in this region. Three other regions had a deficiency of polymorphism (dSTPP, eve, and pSCCH) and were major contributors to the rejection of the neutral model with the HKA test.

**Table 2:** HKA test for the TL gene arrangement

Gene	TL		Dpse *vs.* D mir	
	S Obs	S Exp	D Obs	D Exp
pSTPP	2	2.67	5.28	4.61
en	9	5.35	3.28	6.93
pHYSC	11	7.21	8.68	12.47
exu1	2	1.29	0.96	1.67
pSTAR	10	12.05	22.87	20.83
pHYST	21	15.40	21.03	26.63
dSTPP	0	3.58	9.77	6.19
dSCTL	8	7.98	13.77	13.79
eve	0	1.99	4.58	2.58
Mef2	12	10.52	12.12	13.60
Amy1	13	9.44	8.66	12.22
pSCCH	0	5.02	13.71	8.68
dSTAR	33	22.79	19.28	29.49
dSCCH	2	2.11	3.77	3.66
F6	5	7.72	12.70	9.99
dHYSC	7	6.77	11.48	11.71
dHYST	0	12.67	34.56	21.90
EcR	2	2.43	3.57	3.14
*T*	2.17			
χ^2^	20.91			
*P*	0.047			
Sim	9097			

HKA, Hudson Kreitman, and Aguade; TL, Tree Line; S Obs, observed number of segregating sites; S Exp, expected number of segregating sites; D Obs, observed number of divergent sites; D Exp, expected number of divergent sites; T, Coalescence time between *D. pseudoobscura* and *D. miranda*; χ^2^, HKA test statistic; *P*, probability of a χ^2^ statistic greater than that the observed value; Sim, number of coalescent simulations run.

Given the suggested excesses of polymorphism, it is interesting to consider the distribution of these polymorphisms among the five different arrangements. A unique polymorphism is one that is segregating in one arrangement and not the others. Alternatively, a shared polymorphism is one that is variable in two or more arrangements. One can use the outgroup species *D. miranda* to infer which of the polymorphic nucleotides is the derived mutation. The frequency of derived mutations tends to be greater in breakpoint than in nonbreakpoint regions, and older arrangements have accumulated larger numbers of fixed derived mutations (Figure S3). Of the 758 polymorphic sites in the 18 genetic markers, there was a significant excess of sites that were unique to one arrangement (466 observed *vs.* 258.6 expected) based on the assumption that polymorphisms occur independently on the different arrangements (χ^2^ = 512.7, df = 31, *P* = 3.7 × 10^−91^; see File S1 for a description of the analysis and Figure S4). The youngest arrangement AR has a significant excess of unique polymorphisms compared with the four other arrangements (χ^2^ = 16.5, *df* = 4, *P* = 0.002). TL had an excess of sites (14 observed *vs.* 4.2 expected) that were uniquely fixed within this arrangement (χ^2^ = 30.5, df = 2, *P* = 3.4x10^−7^). Eleven of these sites are fixed for nucleotide changes in the dHYSC gene region. Additional tests show that unique polymorphisms and fixed differences are not homogeneous among arrangements (File S1, Table S8, Table S9, and Table S10).

The results of the three tests for selection (Tajima, coalescent, HKA) reveal a complex evolutionary history for the third chromosome. As has been seen with the majority of loci in *D. pseudoobscura*, Tajima’s *D* is negative for 76.9% of the loci on the third chromosome, which strongly supports a demographic rather than selective explanation for the excess of rare variants seen at most loci, but only four loci showed a significant excess of rare variants. The other 23.1% of the loci show a greater frequency of intermediate variants including two breakpoint loci with significant positive *D* values, which may indicate the selective maintenance of gene arrangements.

The coalescent simulations show levels of heterozygosity fail to reject a neutral model of molecular evolution when a constant population is assumed (Figure 2). When the more realistic exponentially growing population model is used, we observe some loci with significantly more polymorphism within arrangement than expected (Figure 3). This signature suggests that the maintenance of the different arrangements through selection in heterogeneous environments (Schaeffer 2008) has elevated the levels of polymorphism within particular arrangements. The analysis of derived mutations is consistent with this interpretation because there is an excess of unique derived mutations in each of the five arrangements (Table S7). Unique mutations are expected in inversion genealogies because the suppression of recombination will limit the transmission of polymorphisms among different arrangements, but our data suggest that the selective maintenance of the different arrangements has allowed the accumulation of more unique polymorphisms than expected under a neutral model. Although all arrangements show an excess of unique polymorphisms, the AR has more unique polymorphisms than expected given the proportion of segregating sites in AR relative to the total number (Table S7). This is quite striking because the AR is one of the youngest arrangements and has one of the widest distributions within the species. These data suggest that this chromosome has increased at a rate greater than the overall rate of population expansion.

The pattern of nucleotide variation in the TL arrangement suggests a complex evolutionary history compared to the other four arrangements. Four loci within TL (dSTPP, eve, pSCCH, and dHYST) have no segregating sites. This low level of variation could indicate strong selective constraint or a recent selective sweep on a sequence near these four regions. The rejection of the neutral model with the HKA test suggests that these four regions are near a region that has recent selective sweep because the *D. miranda* outgroup sequence is not selectively constrained. The dHYSC region has seven derived variants with a frequency of 0.25, but the region also accounts for 11 of 14 fixed derived mutations in the TL arrangement. This suggests that this region may be recovering from a recent selective sweep. This is consistent with numerical models of selection in heterogeneous environments that find evidence for selective sweeps and balancing selection operating on the inversions (Schaeffer 2008).

### Derived gene arrangements fail to show reduced DNA divergence compared with ancestral gene arrangements

The theoretical work of Navarro *et al.* (1997, 2000) developed predictions about how the past selective history of chromosomal inversions and genetic flux will affect the patterns of nucleotide heterozygosity and differentiation within and outside the inverted regions. We tested the Navarro *et al.* (1997, 2000) predictions by plotting nucleotide heterozygosity for the 11 inversion breakpoint and seven nonbreakpoint regions in the derived, ancestral, and both arrangements combined. We contrasted two sets of data. The first comparison examined the smaller and younger AR chromosome that was derived from ST (ST → AR, 5.9-Mb inversion accounting for ~23.8 cM on the genetic map) and the second comparison observed the larger and older PP arrangement derived from ST (ST → PP, 11.6-Mb inversion accounting for ~46.9 cM on the genetic map).

**Figure 4:**
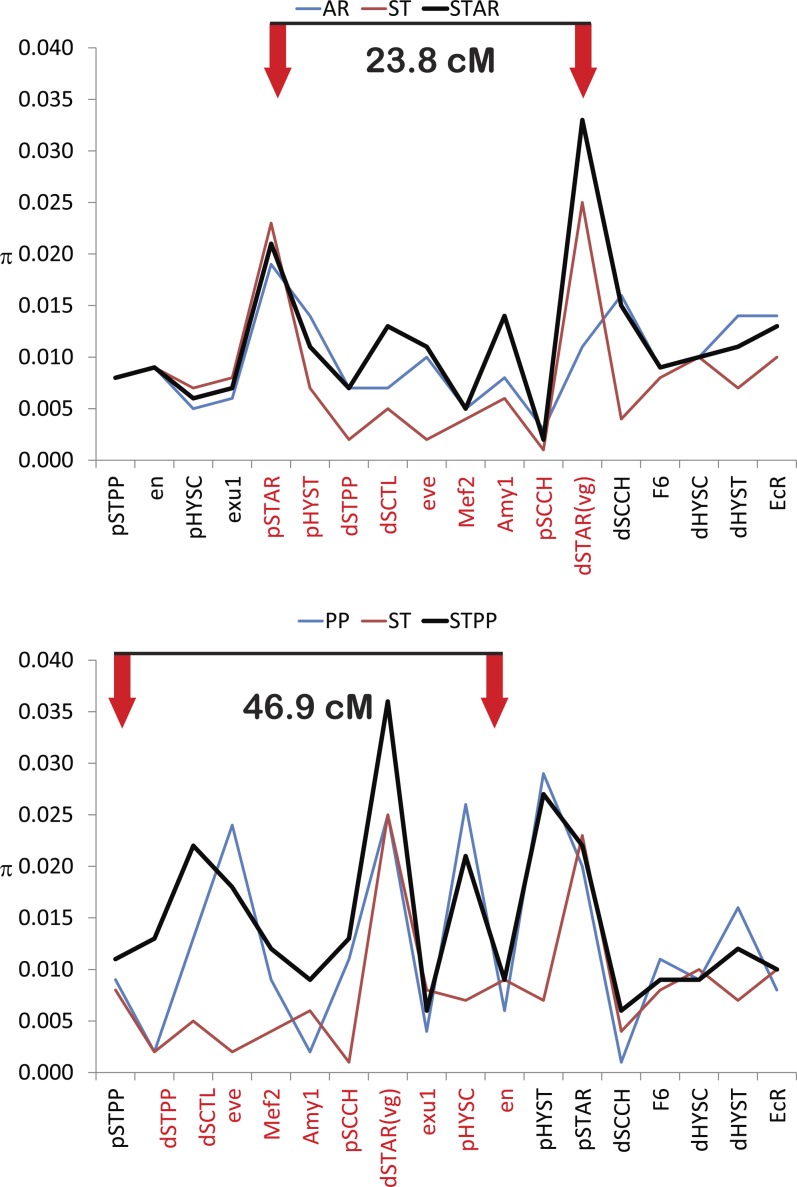
Silent nucleotide heterozygosity (π, Tajima 1983) along the chromosomes in ancestral and derived arrangements of *D. pseudoobscura*. The blue line shows levels of variation within a derived arrangement, the red line shows levels of variation in the ancestral arrangement, and the thick black line shows levels of variation among the ancestral and derived gene arrangements. The two red arrows in each panel show the locations of the proximal and distal breakpoints that converted the ancestral into the derived arrangement. The order of genes in each panel is given according to the derived arrangement. The genetic markers labeled in red on the x-axis are genes within the inverted region of the derived arrangement. The proximal region is to the left.

The more recent event (ST to AR) shows that silent heterozygosity levels are elevated at the two breakpoint regions (red arrows) but are lower in the proximal, central inverted region, and distal regions. The derived AR arrangement has lower heterozygosity levels at its two breakpoint regions compared with the ancestral ST arrangement or randomly chosen AR or ST chromosomes, but in the majority of other regions AR has greater levels of heterozygosity than ST (Figure 4). These data support the predictions of the Navarro *et al.* (2000) model at breakpoints but not in the central regions where heterozygosity in the derived arrangement is greater than in the ancestral arrangement.

The pattern for the ST to PP data provides less support for the Navarro *et al.* (2000) model. The ancestral ST arrangement has lower levels of heterozygosity estimates at 10 of 18 regions. The observed pattern differs from the expected pattern given a neutral coalescent model where the more derived arrangement has consistently lower heterozygosity (Navarro *et al.* 2000).

According to the predictions of Navarro *et al.* (2000), patterns of nucleotide variability along the chromosome of gene arrangements will depend on the age of the inversion. This prediction is based on theory that shows that as new inversions increase in frequency, they can eliminate variability in a large segment of the chromosome by means of a possible selective sweep. The resulting effect is low variability at the breakpoints of new inversions and higher variability within the inverted segment. However, older inversions that have reached equilibrium will show greater variability at the breakpoints than within the inverted segment. The ST/AR comparison is our best test of the Navarro *et al.* (2000) model because the breakpoints have been precisely mapped (Richards *et al.* 2005). The comparison of the ST/AR arrangements shows that nucleotide diversity is elevated at the ST to AR breakpoints with the ancestral ST arrangement having higher levels of diversity than the derived AR chromosome. The elevation of nucleotide heterozygosity at the breakpoints is expected given the age of the ST to AR inversion. This inversion mutation was estimated to have occurred 580,000 years ago (Wallace *et al.* 2011). If we assume that *D. pseudoobscura* has four generations per year (Babcock and Anderson 1996), then AR has existed for more than 1 million generations and would be expected to show elevated levels of nucleotide diversity at the breakpoints as these regions continued to accumulate nucleotide mutations. The proximal, distal, and inverted regions, however, failed to show the derived AR arrangement with consistently lower levels of variation compared to the ancestral ST chromosome (Table 1 and Figure 4). It should be noted, however, that the confidence intervals for heterozygosity in the ST and AR arrangements overlap (Table S2).

The pattern for the ST/PP comparison is quite different from the ST/AR data. The markers closest to the breakpoints do not show an elevation of nucleotide heterozygosity. At this time, we have not precisely mapped the positions of the ST to PP breakpoints so the lack of elevated heterozygosity may not be surprising. Despite this, the markers within the inverted region do not show reduced variability in the derived PP arrangement. In most cases, markers within the ST arrangement have lower heterozygosity than PP. The likely explanation is that ST to PP inversion is twice the size of the AR arrangement, which will likely result in more genetic exchange among chromosomes leading to more independence in the genetic information.

The lack of concordance of the *D. pseudoobscura* data with the predictions from the Navarro *et al.* (1997, 2000) models may reflect that the inversion polymorphism in *D. pseudoobscura* is not a simple two arrangement polymorphism. The arrangements of *D. pseudoobscura* were generated through a set of overlapping inversions generated at different times and the opportunity for genetic exchange between the different inversions depends on the localities where each arrangement is found in nature. The fact that the different arrangements were generated at different times will determine when each chromosome begins to diverge. The pairing of chromosomes separated by two or more inversion mutations will serve to increase recombination suppression. The Navarro *et al.* (2000) model assumes that the derived arrangement has swept to intermediate frequency and the increase in frequency of derived arrangements in *D. pseudoobscura* may not resulted from a simple sweep. Finally, the different arrangements are distributed in a highly structured manner among populations potentially due to local adaptation (Schaeffer 2008) This level of structure will determine which arrangements have the opportunity to pair as heterozygotes (Schaeffer and Anderson 2005). This will not always be with chromosomes that are phylogenetically related. For instance, PP and AR co-occur in Texas, but these two arrangements are separated by two inversion steps. ST and CH co-occur in California, but these two arrangements are separated by three inversion steps.

### How recombination suppression structures genetic variation in the presence of chromosomal rearrangements

Reduced recombination among the gene arrangements is expected to result in LD or nonrandom associations among nucleotide sites. Patterns of LD can also result from population structure and balancing selection. Pairs of segregating sites across all gene arrangements were tested for significant LD.

**Figure 5:**
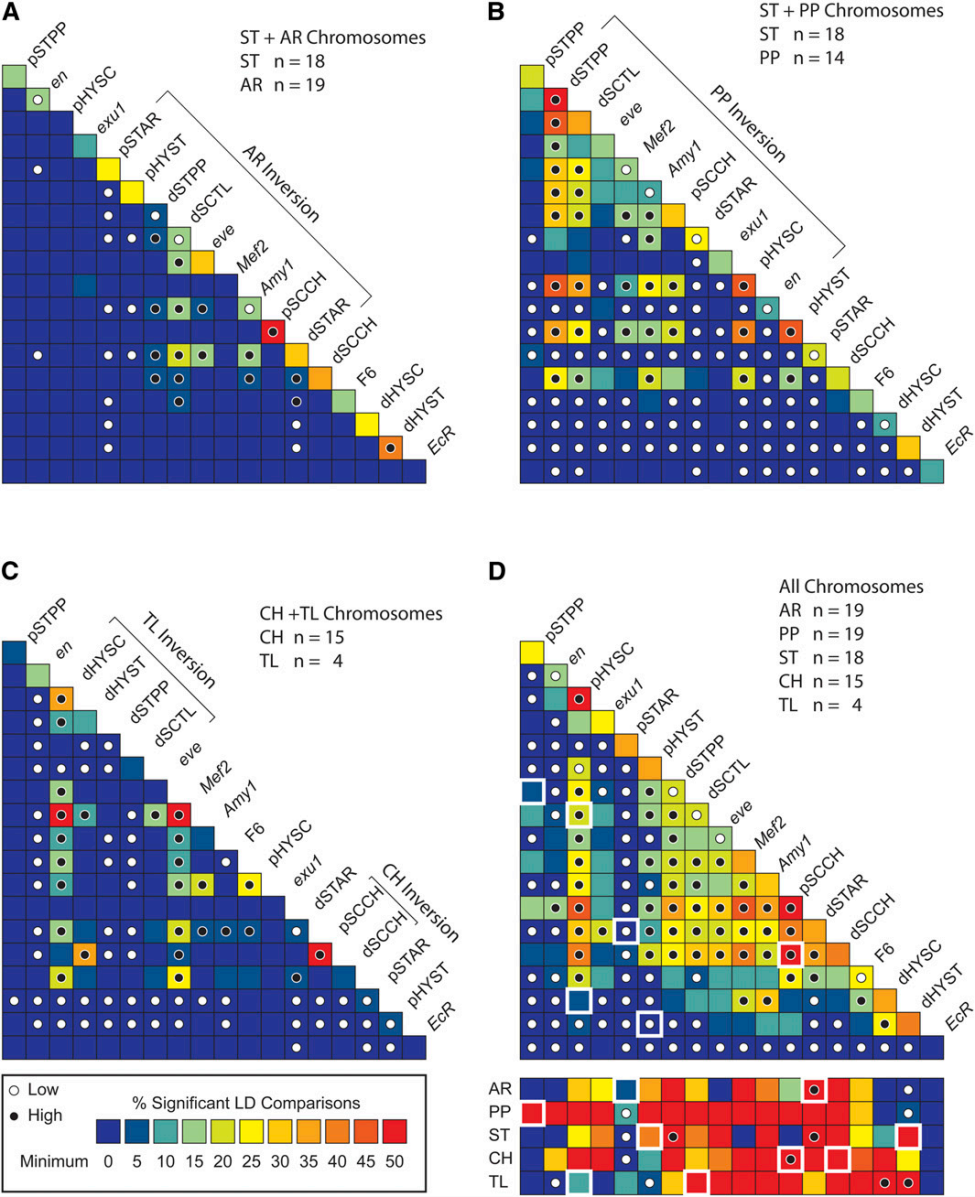
Pairwise LD plots for within- and between-locus comparisons across the third chromosome of *D. pseudoobscura*. Each block in the matrix presents the fraction of pairwise comparisons of sites that were in significant LD. In all plots, levels of LD within or between loci are represented as percentage of valid comparisons that were less than the respective significance cutoff using a false-discovery rate of 1% (Storey 2002). For (A−C), the loci within the inverted segment are indicated by the bracket. The key to the heat map is shown at the bottom left hand side of the figure where lower levels of association are marked in blue and higher levels of association are redder. White dots indicate within or between locus comparisons that were significantly lower than the average % of significant comparisons, whereas the black dots indicate comparisons that were significantly greater than the average % significant comparisons. (A) Heat map for LD using the ST and AR chromosomes. Significance cutoff was *P* = 0.00195. The loci (top to bottom) are given in the order of the Arrowhead arrangement. The average percentage of significant comparisons within locus was 27.4% (432 of 1574) and the average percentage of significant comparisons between loci was 2.0% (371 of 18,334). (B) Heat map for LD using the ST and PP chromosomes. Significance cutoff was *P* = 0.00195. The loci (top to bottom) are given in the order of the PP arrangement. The average percentage of significant comparisons within locus was 34.8% (706 of 2030) and the average percentage of significant comparisons between loci was 8.0% (1996 of 24,829). (C) Heat map for LD using the CH and TL chromosomes. Significance cutoff was *P* = 0.0029. The loci (top to bottom) are given in the order of the CH arrangement. The average percentage of significant comparisons within locus was 15.1% (316 of 2087) and the average percentage of significant comparisons between loci was 4.3% (943 of 21896). (D) Heat map for LD for all *D. pseudoobscura* chromosomes. Significance cutoff was *P* = 0.0047. The loci (top to bottom) are given in the order of the Arrowhead arrangement. The box below the triangular matrix shows significant associations of nucleotide sites within a gene locus with the corresponding gene arrangement shown in rows. The significance cutoff for these tests was *P* = 0.021. The interlocus boxes highlighted in white indicate comparisons nucleotides between the proximal and distal breakpoints. The average percentage of significant comparisons within locus was 35.3% (1385 of 3922), and the average percentage of significant comparisons between loci was 11.3% (5808 of 51,573).

We first examined LD among nucleotide sites across all arrangements. A total of 54,911 of the possible 2,249,006 pairwise comparisons of the 671 segregating sites were capable of rejecting the null hypothesis of no association with Fisher’s exact test. Intralocus and interlocus LD for the 18 genetic markers were estimated as the percentage of site pairs that had a significant LD value (Figure 5D). A general result is that the central region of the third chromosome tends to have highest levels of LD than centromeric and telomeric regions. There are hot and cold spots for LD across the chromosome with both random association among adjacent regions and nonrandom associations between distant sites (Figure 5D), a pattern previously observed by Schaeffer *et al.* (2003).

Breakpoint regions tended to have higher levels of LD than nonbreakpoint regions. Breakpoint regions had a higher fraction of site pairs that showed significant nonrandom associations within locus than non-breakpoint regions (39.8% *vs.* 22.8%, respectively). Interlocus comparisons of breakpoint regions also had greater fractions of site pairs in significant LD than interlocus comparisons of nonbreakpoint regions (14.5% *vs.* 8.1%, respectively).

We asked whether the proximal and distal breakpoints for the five gene arrangements were in LD with each other. One might expect nucleotide variation at the two ends of an inversion to show significant association because allelic variation initially captured by the original inversion event will be associated along the entire inverted region and the strong suppression of recombination at the breakpoints will maintain these associations. Two pairs of inversion breakpoints support this hypothesis, the SC to TL and the SC to CH inversions, with 22.3 and 55.4% of the sites pairs in significant LD. The other four pairs of inversion breakpoints had few nonrandom associations despite these arrangements resulting from older inversion events.

LD increases as a gene arrangement’s age increases. The AR and PP arrangements are each derived from the ST arrangement. We examined the pattern of significant LD estimated within and between loci for the younger comparison of ST and AR chromosomes and the older comparison of ST and PP arrangements. The ST and AR comparisons had lower levels of LD within and between loci than the ST and PP comparisons (Figures 5, A and B). We can rule out differences in sample size as a potential explanation for why the AR/ST comparisons had fewer significant nonrandom associations than the PP/ST comparisons. The AR sample size was greater than that of PP, yet had fewer significant test results. In both cases, the greatest interlocus LD was observed among loci within the inversion breakpoints. We tested for significant LD for TL and CH chromosomes, which are both derived from the SC arrangement, and found a modest level of nonrandom associations among sites within and between loci (Figure 5C). Interlocus LD appeared to be lower for the markers closest to the ends of the chromosome than for markers in the center of the chromosome. The dHYST and EcR markers are closest to the distal end of the chromosome and showed low levels of interlocus LD whereas the pSTPP and *en* markers closest to the proximal end of the chromosome showed low interlocus LD. All other markers positioned on other areas of the chromosome have higher levels of interlocus LD with the exception of the pSTAR region that showed no interlocus LD.

Variation at the segregating sites showed significant associations with gene arrangement. LD was estimated between nucleotide sites and the five different gene arrangements (AR, PP, ST, CH and TL). A total of 944 of the possible 3355 pairwise comparisons of the 671 segregating sites were capable of rejecting the null hypothesis of no association between the segregating site and the inversion type with Fisher’s exact test. It was shown that the PP arrangement had the highest levels of LD and the AR arrangement had the lowest levels of LD. The EcR locus showed no evidence of LD in any of the gene arrangements. The pSTPP marker only showed evidence of LD with the PP arrangement. The markers at the extreme end of the chromosome showed no evidence of LD with the arrangements with the exception of the pSTPP breakpoint region in the PP arrangement. The PP arrangement had the longest continuous segment of LD along the chromosome.

Over time, chromosomal rearrangements will differentiate from one another and accumulate unique polymorphisms because recombination suppression will isolate the different gene arrangements from each other. This predicts that LD will increase with gene arrangement age. This is what was observed. When the ST sequences were combined with the younger AR sequences, we saw modest levels of LD within locus and relatively low levels of LD among loci. When the ST sequences were combined with older PP sequences, we saw higher levels of LD within and between loci.

An unusual feature of the LD analysis was that pairs of breakpoints for a single arrangement, say pSTAR and dSTAR, did not show high levels of LD. This was an unexpected result because one might expect that the initial inversion mutation would create LD among the polymorphic sites at the proximal and distal boundary regions and these associations would be maintained over time. For some breakpoints in our study, we are not confident that the genetic markers used were absolutely adjacent to the breakpoint regions. The ST to AR inversion breakpoints, however, have been precisely mapped and these genetic markers (pSTAR and dSTAR) are within 0.5 and 5.6 kb of their respective breakpoints. These markers show a deficiency of LD between the two breakpoints, despite high levels of intralocus LD (Figure 5D). This finding suggests that the regions near the inversion breakpoints did not capture segregating allelic sites at the time of the original inversion mutation. It appears that all LD associated with these two inversion breakpoints has accumulated since the inversion mutation happened. The distance that separates the two breakpoints is sufficient to allow these regions to evolve independently suggesting that new nucleotide mutations do not co-occur on the same chromosome.

The results of the LD analysis within each arrangement provide a link between estimates of LD and the length of inversions. The PP arrangement had significant levels of LD distributed over a large portion of the chromosome (Figure 5). In fact, the pSTPP marker only showed evidence of LD in the PP arrangement, and it is close to the proximal breakpoint region of the PP chromosome. This finding is concordant with the model that newer inversions have low rates of recombination near the inversion breakpoints and low rates of recombination are indicated by significant LD. The breakpoint regions within the other gene arrangements also show significant LD, except for the AR arrangement. Overall, inversion breakpoint regions tend to provide more evidence of LD than non-breakpoint regions. In addition to estimating rates of nucleotide diversity, inversion breakpoints may provide information regarding rates of recombination by estimates of LD.

The central region of the chromosome harbors the greatest levels of LD despite the expectations of the Navarro *et al.* (1997) model, which suggests that genetic flux will be greatest in the central regions of inversions. Our data may differ from this expectation because we are dealing with a system of overlapping inversion mutations. The central region of the chromosome is expected to be inverted in most gene arrangement heterozygote combinations. Thus, the central regions would be expected to experience the strongest amount of recombination suppression compared to either the proximal or distal regions. The reduced recombination leads to strong non-random associations between sites. Thus, a system of overlapping inversions can increase the level of genetic structure and severely reduce genetic flux over a wider region of the chromosome.

Models of Navarro *et al.* (1997, 2000) made predictions about how genetic flux in the presence of paracentric inversions will impact genetic diversity along chromosomes. Our data did not conclusively support the predictions of these models. In some cases, we found that variation was elevated near some breakpoints, but not in others. Older inversions fail to show greater levels of diversity than derived mutations, even though breakpoint regions accumulate unique mutations, which should elevate levels of diversity and divergence. Breakpoint regions show elevated levels of LD, yet segregating sites within pairs of breakpoints fail to show nonrandom association with each other. The observed decoupling of genetic diversity patterns across the third chromosome of *D. pseudoobscura* reveals a complex history that reflects neutral and potentially selective forces acting in nature. Future analysis of the third chromosome with next generation re-sequencing will help to clarify issues left unanswered in this work.

## Supplementary Material

Supporting Information
