# Supplementary material for: Molecular Population Genetics of Inversion Breakpoint Regions in Drosophila pseudoobscura
Source: G3 (Bethesda). 2013 Jul 1;3(7):1151–63. doi: 10.1534/g3.113.006122 (PMC3704243; doi:10.1534/g3.113.006122)
Supplement: Supporting Information [file supp_3_7_1151__index.html]

Molecular Population Genetics of Inversion Breakpoint Regions in Drosophila pseudoobscura — Supporting Information 

# Molecular Population Genetics of Inversion Breakpoint Regions in *Drosophila pseudoobscura*

## Supporting Information for Wallace, Detweiler, and Schaeffer, 2013

**Files in this Data Supplement:**

- Supporting Information - Figures S1-S4, Files S1-S2, and Tables S1-S10 (PDF, 2 MB)
- Figure S1 - Observed versus expected estimates of nucleotide heterozygosity (Θw) at 18 *D. pseudoobscura* marker loci based on a coalescent analysis of a population of constant size (PDF, 518 KB)
- Figure S2 - Observed versus expected estimates of nucleotide heterozygosity (Θw) at 18 *D. pseudoobscura* marker loci based on a coalescent model of an exponentially growing population (PDF, 503 KB)
- Figure S3 - Frequency of variants at the segregating sites in the five gene arrangements (PDF, 478 KB)
- Figure S4 - Frequency spectra of derived mutations in breakpoint and non-breakpoint regions for five gene arrangements of *Drosophila pseudoobscura* (PDF, 983 KB)
- File S1 - Supporting Data (PDF, 184 KB)
- Table S1 - Primer Sequences Used for PCR Amplification (PDF, 99 KB)
- Table S2 - Nucleotide Polymorphism in 18 Regions of the *D. pseudoobscura* Third Chromosome (PDF, 109 KB)
- Table S3 - HKA test for the Arrowhead gene arrangement (PDF, 55 KB)
- File S2 - Literature Cited (PDF, 214 KB)
- Table S4 - HKA test for the Pikes Peak gene arrangement (PDF, 61 KB)
- Table S5 - HKA test for the Standard gene arrangement (PDF, 54 KB)
- Table S6 - HKA test for the Chiracahua gene arrangement (PDF, 54 KB)
- Table S7 - Shared, unique, and fixed polymorphisms among the five gene arrangements of *D. pseudoobscura* (PDF, 69 KB)
- Table S8 - Observed and expected numbers of polymorphic sites classified into the six unique, shared, and fixed categories for the five gene arrangements of *D. pseudoobscura* (PDF, 65 KB)
- Table S9 - Observed and (expected) numbers of unique polymorphic sites for the five gene arrangements of *D. pseudoobscura* (PDF, 64 KB)
- Table S10 - Observed and (expected) numbers of category 0 polymorphic sites for the three gene arrangements of *D. pseudoobscura* (PDF, 64 KB)
