# Supplementary material for: Molecular Population Genetics of Inversion Breakpoint Regions in Drosophila pseudoobscura
Source: G3 (Bethesda). 2013 Jul 1;3(7):1151–63. doi: 10.1534/g3.113.006122 (PMC3704243; doi:10.1534/g3.113.006122)
Supplement: Supporting Information [file supp_g3.113.006122_006122SIf.pdf]

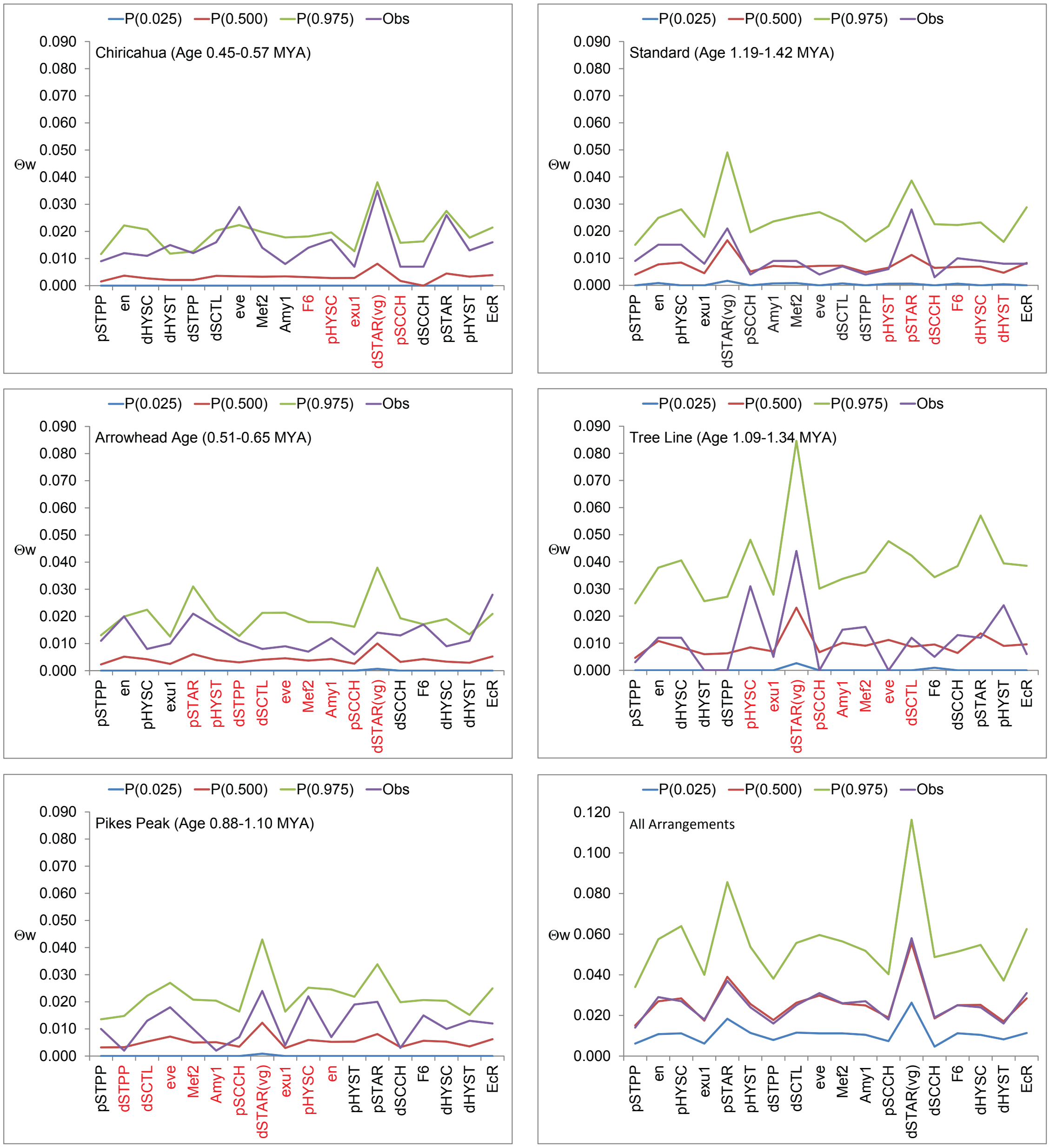

Figure S1 Observed versus expected estimates of nucleotide heterozygosity (Θw) at 18 *D. pseudoobscura* marker loci based on a coalescent analysis of a population of constant size. Nucleotide heterozygosity estimates based on the number of segregating sites (Θw) within five gene arrangements (AR, PP, CH, ST, and TL) as well as among all arrangements are shown in the individual panels. The observed (Obs) estimates of Θw as well as the mean P(0.500) and the 95% confidence interval (P(0.025) to P(0.975)) derived from 1000 coalescent simulations using nested subsamples for a constant population size model. The genetic markers labeled in red on the x-axis are genes within the inverted region of the derived arrangement. The proximal region is to the left.

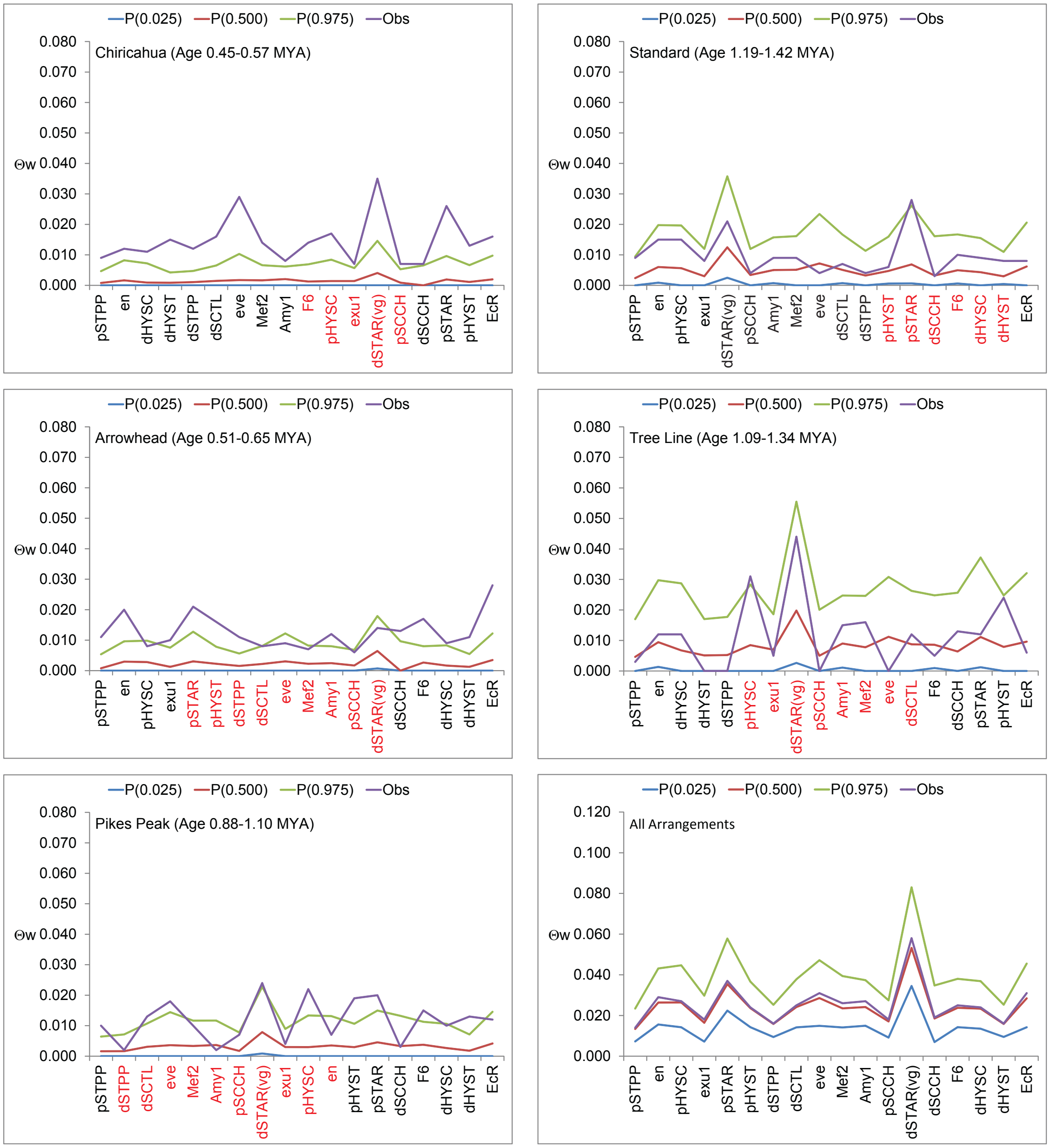

Figure S2 Observed versus expected estimates of nucleotide heterozygosity (Θw) at 18 *D. pseudoobscura* marker loci based on a coalescent model of an exponentially growing population. Nucleotide heterozygosity estimates based on the number of segregating sites within five gene arrangements (AR, PP, CH, ST, and TL) as well as among all arrangements are shown in the individual panels. The observed (Obs) estimates of Θw as well as the mean (P(0.500) and the 95% confidence interval (P(0.025) to P(0.975)) derived from 1000 coalescent simulations using nested subsamples for an exponentially growing population with growth rate parameter α=7. The genetic markers labeled in red on the x-axis are genes within the inverted region of the derived arrangement. The proximal region is to the left.

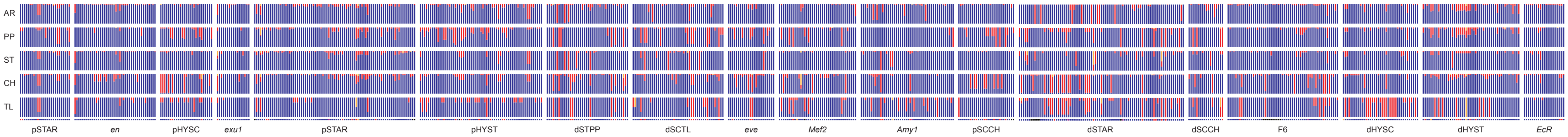

Figure S3 Frequency of variants at the segregating sites in the five gene arrangements. The five bars represent the frequencies of variants in the five gene arrangements. Each column represents a single segregating site. For each site, the colors of segregating bases from most frequent to least frequent in decreasing order are: blue, red, orange, and purple. The bottom line indicates the base of *D. miranda*, where an indel is designated by a black square. The segregating sites of the different gene regions are separated by white space.

Breakpoint Regions

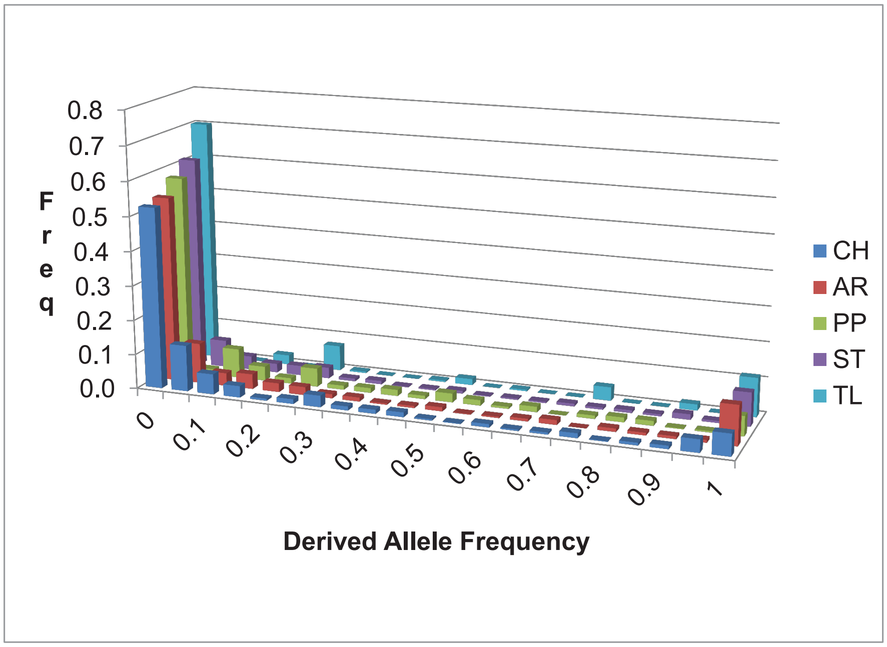

Non-Breakpoint Regions

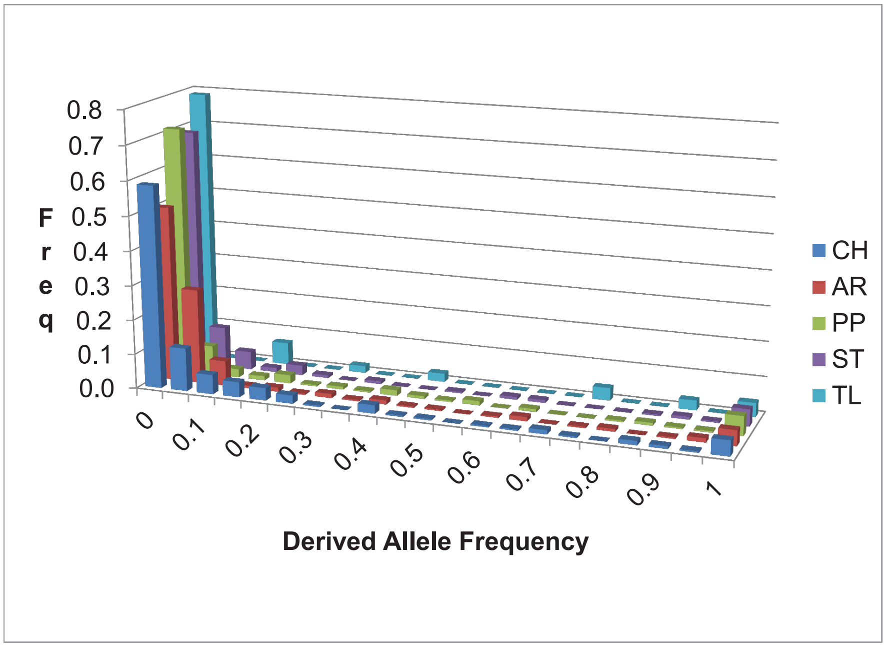

Figure S4 Frequency spectra of derived mutations in breakpoint and non-breakpoint regions for five gene arrangements of *Drosophila pseudoobscura*. A total of 509 and 249 segregating sites were in breakpoint and non-breakpoint regions, respectively. The relative frequency of sites is displayed on the z-axis, which is the number of sites that have a particular site frequency divided by the total number of sites. This allows the frequency spectra of the two types of regions to be compared. The arrangements are ordered on the y-axis from youngest (CH in front) to oldest (TL in back).

File S1

Supporting Data

***D. pseudoobscura* Inversion Gene Markers**

The following list provides the sequences of the 18 gene markers in the *D. pseudoobscura* reference strains MV2-25; (RICHARDS *et al.* 2005) used in this study. The title shown in bold gives the number of the marker, the name of the marker, the reference genome strain, the length of the amplified region excluding primers in the reference genome, the length of the aligned sequence. The coordinates for the FASTA entry are given on second line based on release (R2.27) in FlyBase (http://flybase.org) where 3 indicates chromosome 3 of *D. pseudoobscura*. The noncoding sequence is shown in lower case letters and coding sequence is indicated in upper case letters. Coding information is shown below the sequence.

01 pSTPP, MV2-25
Ref Length=354 bp, Aligned Length= 363 bp, Silent Sites w/o-Gaps = 353 bp

```
>3:2292752,2293105
gacgaaaccaaaacctaaacagaactcattttgattcacataacagtgggctgtccgcgg
tctggaaaccatcatttagcatcaaacgtaccaataaaatataataataaccaaacttat
caaagcgaatgttaattagtgcggaaattcacactaatctgcgtgcttccaggctcgttt
ccatgtaattcgtttatccatcagtgccatctatctattccgccgcaaatgggcatttaa
ttaatgtccaaattcgattttagacaaatgtttatccatcacatcaatacaaatctaaat
tgtgcgtgtttgcggtatcctcgcacttgccagcacattttacctttgacgtta
```

02 engrailed, MV2-25
Ref Length=376 bp, Aligned Length= 407 bp, , Silent Sites w/o Gaps = 302 bp

```
>3:4595297,4595673 (reverse complemented)
cacacaagcactcacacagacccatgtagaagccatagatcccttgttgtgtcatatttg
caccaaaatatttacacatattttccgagacccccgcggcgattgcatcctggcaaacgc
atgtcctgcctcggtccccggtcccctgtccccggtccgtacttaaccaattagacacgc
tcatatctcacccggacttccatctactggcagtctgtcttcggggtgcctctttcaact
caaaccgcaaataattgatttgccttacctacctaccagcctcccatagtagatcccatc
cgtatctctgtgcctctgtactcatcccagactcctctctgatctttctacttgcagCGG
GAGTTCAACGAGAATCG
```

Coding information GA21479

Region	Alignment Coordinates	Actual Coordinates
Intron 1	1..386	4595645..4955258
Exon 2 (position 1)	387..407	4955257..4595297

03 pHYSC, MV2-25
Ref Length=375 bp, Aligned Length= 398 bp, , Silent Sites w/o Gaps = 193 bp

```
>3:6432216,6432591
tatttggacgcagactcagtttaattttcacattttagcccaaaattcactcctagggcc
ccgggcagacaaaggcagacaaactgcgctgcaaccgaataaaccatttacaaatacgag
tagcattcgagcaaatataggcgattggcattctatttgaatgagctgcatggagcgtcg
gtcttaagcttaaggcaattgtttgagtgccttttgctgccgttgatggataggattcaa
atagccagaggcgtgggatcacgtaatgcacttccccagcaaccactgggccacaaaaac
gaggcagcaaataaggcagtggccaaagtcagccactaattgcaacattaaagctccttt
tggttgccactgtgtg
```

04 exuperantia 1, MV2-25
Ref Length=356 bp, Aligned Length= 358 bp, Silent Sites w/o-Gaps= 176 bp

```
>3:8591272,8591628
gggcattgaccctctgcgtcggacctaacctaacttttctctacgtctcccctttgccca
ttttccagATTGTCCAGTTGGCTGCCTACACTCCAAAGGACAACTTCCAGCAGTACATCA
TGCCGTATATGAATCTGAATCCAGCCGCTCGTCAGCGTCATCAAATTCGTGTGATTTCGA
TCGGCTTTTATCGCATGCTGAAGTCGATGCAGACCTACAAGgtacattcatacctacact
cccgttatgtccgctaattctagcttttattttctcagATTATCAAATCTAAATCGGAGG
TTGCTGCTCTCATGGACTTTCTCAACTGGCTTGAGATGCTGGTCGCCAAACAGCCCA
```

Coding Information GA21461

Region	Alignment Coordinates	Actual Coordinates
Intron 1	1.. 68	8591272..8591339
Exon 2 (position 1)	69..221	8591340..8591492
Intron 2	222..279	8591493..8591550
Exon 3 (position 1)	280..358	8591551..8591628

05 pSTAR, MV2-25
Ref Length= 467 bp, Aligned Length= 473 bp, Silent Sites w/o-Gaps= 440 bp

```
>3:8900356,8900823
gatgacaaacatattcttaattctataacatccattcccccagggtatgtgtgcgtggaa
tgggactcattgttgtgaaccggttatgacgactaattcttggttaatctttcttccgtc
gaatattcccagctatatagatctcttatttttgcccagataatttaatatgatagctgt
atcaattttgatcaagattaactatttttaaatactcgcatgtttttttgttttgacaaaa
acacgatttcatcaaaaatgttcaatgccgcaggttgtgacgtcaatgttgctggtattt
gcagattcttttgttgtcaaccagggtatgagcgtttgtataccctatttcgtgaatatt
atataaagatactgttttccaagctgctttcaaatgtaattaataaaggccttgttttag
attgcatttttttggtctattcatgcatttgattagttatttgtatat
```

06 pHYST, MV2-25
Ref Length= 805 bp, Aligned Length= 1,486 bp, Silent Sites w/o-Gaps= 484 bp

```
>3:9140888,9141693
CGCAATGCAAAGCCATGTCGATATTGAAGAATTCTTTATGAAAGTGGATTCAAGTGGTGC
GCACGATCAGACGGTGCACGgtaagtttatagcatatacatttgtgtatattcgctcata
tgctttcttttttgatagGTCTTGGCGTATACGATTTGCACGAAATGGTGGAAAAGGCTAA
GAAGATAGATCAACAAATGCAGTCCTCTCAGCCCACCTTTATCCATCATCGCTTTCGCTA
Actgcctttgaaaaactttaagactataacaacaacaaaattattattattcatgtcatt
atttttggatatgcttattgccttactttgttcgattgttcggttatttggaaaaatttc
aatattgtttattgcttctgtcccgggtaccctgtgattatctatttacgattcagtggc
acttcgataagaagccacaaaagcgaaattaaagcaagaatggattgtgcagaagatcgg
atatctttataactatagtttattagtgttcaatcagcgtttagagtaattaaagcaatt
taactctaccatgcataagtgaacacagcctaaaataacacgttcgtgtcattcaaattt
tattgtgctctcaagagcaggaacaatgcgtggccagctgattatttctgtgcttctcgg
attattcctaacattccacatcaatataaacgtaaatataatactagtaaagtggacaat
attgaaccaaatttttagGATGCCGTCGTCTTTAAATTTACAAACGTCGTCTGCGGGAGT
TACAACCAATCCTGGTTCGTGTTCCA
```

Coding Information GA21596 (Dmel CG9183) and GA24829

Region	Alignment Coordinates	Actual Coordinates
Exon 1 (position 3)	1.. 80	9140888.. 9140967
Intron	81.. 145	9140968.. 9141024
Exon 2 (position 2)	146.. 247	9141025.. 9141128
Noncoding	248..1418	9141129.. 9141626
Exon 1 (position 1)	1419..1486	9141627.. 9141693

07 dSTPP, MV2-25
Ref Length= 572 bp, Aligned Length= 587 bp, Silent Sites w/o-Gaps= 523 bp

```
>3:9832256,9832828
GTGGAAAGGGTCCTCAAGTAGactctattctactgaaattttacagctttccaggataaa
aaacgatgtagtttttgttctttttctgactttgttttatacaaaaatgcatctttattc
gtttaattattaaatatacacaagaaatctcatatgttcgtttcttttgcatgttttcca
ttttctcatctcgttttgggatttcctcagctaaaaattaaatacaatgcttatttatat
gaatgtgtgaatgcgttaatggtcatagatgtgatcgagattgtgtgcggtgtgtgacgg
tgtgtgcgtggactaacacaattgaggcgcttaaacaaaacaaacggcaaaaaatacaaa
atggagcacgtggcatagagacagtggtggagataacagaacagctggtagggaggaacg
gagcgcgcctggctatggcaaaaatggcgggcgctccactagaggggggatagacataga
gacgagcgggatagagatagatggagtaagacagtcactgtcaaactgttgaggaaccgc
tgcccctggcatcggacttaatactcggccaggg
```

Coding Information GA24854

Region	Alignment Coordinates	Actual Coordinates
Exon (position 1)	1.. 18	9832255..9832275
Noncoding	19..587	9832276..9832828

08 dSCTL, MV2-25

Ref Length= 403 bp, Aligned Length= 444 bp, Silent Sites w/o-Gaps= 374 bp

```
>3:10830478,10830881
gcgtgccaaagtgccaatggcgttttaatttgaagctggagttcatgtcggcgtcagctg
ccggcacagactgggtatcgtttatgagctgccgactgctgttgacttttatttatttaa
tgtttcggctttagtgttttgttttttttgctttttataattttatttggcttttcctgcc
ttggacagagcagagtacgaacctgttcccgacctggctacctgcaaaaacagcaccgag
tggaagatcgatgcaaaactggattaagtccctttttacgccctaatccgcactggacctg
gccctagccctggactcgggtcatcgtgtaatggctaaaagagtgagaaagagaggccga
gtggtagtacgagcgagagagagagagagagcaggcgaatcctgcc
```

09 even-skipped

MV2-25, Ref Length= 373 bp, Aligned Length= 374 bp, Silent Sites w/o-Gaps= 146 bp

```
>3:10904241,10904614 (reverse complemented)
AATCACCATCACGACTCCAATGCCGTGGACCAGAAGCCCCTCGTGGTCGATCTCATGGCC
ACGCAATACGGCAAACCCCAGACGCCGCCGCCTTCTCCAAATGgtaggtttccgcaaagc
ccaatggacacattcgacagattattaacccagcctttaccctcttcttgcagAGTGCCT
CTCCAGTCCGGACAACTCGCTGAACGGCAGCCGCAACTCCGAGATTCCCGCTGATCCGTC
GGTTCGACGCTACCGAACCGCCTTCACACGCGATCAGCTTGGACGTCTCGAGAAGGAGTT
CTACAAGGAAAACTACGTGTCCCGCCCGCGTCGCTGCGAGCTTGCCGCCCAGCTCAACCT
GCCGGAATCCACCA
```

Coding Information GA15349

Region	Alignment Coordinates	Actual Coordinates
Exon 1 (position 1)	1..103	10904614..10904512
Intron 1	104..173	10904511..10904442
Exon 2 (position 2)	174..374	10904441..10904241

10 Myocyte enhancing factor 2
MV2-25, Ref Length= 384 bp, Aligned Length= 413 bp, Silent Sites w/o-Gaps= 315 bp

```
>3:10967159,10967543
CAGGTGGGATGTCCTTGATAAgttagtacagtgtctctggcagaagggtgcacacacatt
tttctatctatcagttgttattgttatcgttatcgttatcgttctcgttctcgttcgatt
tttcgttttattttattcttttgcatgatttcccactgccatccccgcccccacccttaa
aaacttctaacagagaatgtgttttgtgcttatttttgtttgtttttgatttccctcgac
tcacccgccatgaatctaacaccagagttccacaattccaatatacatacatatatagtg
cttcgatctaaggtctaaacgtatctgtgcattttcttatcttcttatcgattggtgcca
tcagTATATCCATCGGGTTCGATGC
```

Coding Information GA12881

Region	Alignment Coordinates	Actual Coordinates
Exon 3 (position 2)	1.. 21	10904614..10904512
Intron 3	22..392	10904511..10904442
Exon 4 (position 2)	393..413	10904441..10904241

11 Amylase 1, MV2-25
Ref Length= 422 bp, Aligned Length= 453 bp, Silent Sites w/o-Gaps= 326 bp

```
>3:11849147,11849569 (reverse complemented)
CTCCGCTGACGATGGTGTCCTGGCCATTCATGTCAACGCCATGTTGTAAggaactcagta
tgtgagaccccaacaaccaatcgagattattactattaaatacacaatgatattatgaaa
tgctcgttgactctgagttgggactgctggaaaggataaagggttagggcaggtgcctgg
gcgtttccagatggtcagtgatatccgtattgtatggcgccaagttgtagagaagcctcc
attcaatccggaagggacgagcagtggggaacgtggaaattaattaaatgctggacaaca
aaggaaagagatgggtcactgcagataagaggcggtggcagatgccaactacaggtgaac
tgaggtggctgtacaacgtgaatagtacgagcgtcgtgtatcgtgcgtcaaacaaggtcc
ccc
```

Coding Information GA24265

Region	Alignment Coordinates	Actual Coordinates
Exon 2 (position 3)	1.. 46	11849569..11849524
Noncoding	47..453	11849520..11849147

12 pSCCH, MV2-25
Ref Length= 378 bp, Aligned Length= 402 bp, Silent Sites w/o-Gaps= 326 bp

```
>3:14259606,14259984
cattccaactgcaacacagcattggtccattagccccaatttgctgggaatgtgggagct
aatgggagtggcggtggccgtacgtgaggagaggcaggcaggcgagcaaagagctcgggg
ttattgccacaggttgttgctggccagagggagagcacaattataatttttttaatctaaa
cccgatgacatgatcgaaaaactactctttttacggcaattaaaattgtaatctaaatat
gatgataaataatgatacaaaaaatgactagagcattgtaaatgttcattagtgcgtttt
atatatccaaactaatacttaagtcccaagagtatttgtttttcaaaaatatctgcctga
ccctgctcttctctttagc
```

13 dSTAR, MV2-25
Ref Length= 516 bp, Aligned Length= 651 bp, Silent Sites w/o-Gaps= 309 bp

```
>3:14801829,14802345
gagacacgcattgtttctctctctctctgtcgatctctgtctgtggcttgtttacattag
tatctggctgaaattgccggtatagtccagataacagtattcataattcttataagatcg
ttgagaaaacttatctagtagccccagcagcacaaaactttcttctattacgctggaatt
tcaaaaatttcatttgatgttggtactttagtggaggcctgcaagccaatatctagatgt
cgatatatgtatatatcatagcagtagtttttggcaacgtcactcatagaaagagagaga
gaaagcaccagtggataccatttcacactcgcgctaattactttacaaattctttgaatt
gcggacgggcactcgcattccaaaccagttaacattcaataaatgatatcaaattttata
aacccgtacccgtaatatccgtactgattgtgaaatatttctccgatttcgctttttagC
ACGTTTGGTGGGCTCCCAACAGCACCAGCATCACAAC
```

Coding Information GA17716

Region	Alignment Coordinates	Actual Coordinates
Intron	1..613	14801829..14802227
Exon (position 2)	614..651	14802228..14802345

14 dSCCH, MV2-25
Ref Length= 353 bp, Aligned Length= 354 bp, Silent Sites w/o-Gaps= 85 bp

```
>3:15426293,15426646 (reverse complemented)
CATCAGTTCCAGGCGGTGCTCCTGGCCCTCAGGACTCTCCAAAGGCGCAAAGGTGGAAAA
CAGAAGGCGAAGGATCTTCGCCGCATAGCAGCCCTGCATTTGGACACATATCACCTGGCC
AGGCACTTCTTTGGCCTGTACGATGTTGCCAATGCAATGTTGTTTATCAATATGTGTGTG
ACCACTACTAGCATCCTGTACCATGCCGTGCAGTACAGGAACCAGTCAATCCCATCCGAC
GGCTGGGGTAACCTCTTTGGCAGTGGCCTTGTTTTGTTCAACTTGTGCGGGACTCTGATG
CTCATGGAAAAGCTGGATCGAGTGGTCAGCTCGTGCAATGTGGGCCCGGCCCTT
```

Coding Information Ga12328

Region	Alignment Coordinates	Actual Coordinates
Exon 1 (position 1)	1..354	15426293..15426646

15 F6, MV2-25
Ref Length= 430 bp, Aligned Length= 434 bp, Silent Sites w/o-Gaps= 428 bp

```
>3:16605742,16606172 (reverse complemented)
TTCATggtttgaaataccaactggagagagaactgtataaactgaactgaataagacctt
tagccacgctctgacagctagaacaaaagccatttgatagggctcccacgacaATGCTTC
TCCGGCACTCGTTGGCGTTGGCCATGTTCCGGCTGGCACTGATCTTTGGGCTGTATCTTC
CCCTATATTCCCCTATCTCTGGCAGTTCCATCTGCGGCAGGAACGAGGTGCAGGTAGCCT
GTGGCAATCCCTGCCCCAAGTCCTGTTATCCCCAACGTTGTGTGGACGTCTTGTGCTATG
GCCGTTGCAACTGCATCGGTGGATACAGACGGGTTAACAAGTACCAGGGGCCCTGTGTCC
TTCCCAGCGAGTGTGGAAGGTATCCCATGTTGGAGATACACAAGCAAACGACCAACAAAG
GAAGGAGCAAA
```

Coding Information

Region	Alignment Coordinates	Actual Coordinates
Exon 1 (position 1) GA24156 comp	1.. 5	16605742..16605746
Noncoding	6.. 116	16605747..16605839
Exon 1 (position 1) GA25052	117..434	16605840..16606172

This is the gene annotation in FlyBase for *D. pseudoobscura*. The pattern of nucleotide diversity is not consistent with a coding region. The dn/ds ratio for the partial sequence is fairly high and the outgroup *D. miranda* sequence has a partial deletion within the gene. The AUG start codon in gene GA25052 is polymorphic (AUG versus UUG). Therefore, we assume the following annotation.

```
>3:16605742,16606172
tttgctccttcctttgttggtcgtttgcttgtgtatctccaacatgggataccttccaca
ctcgctgggaaggacacagggcccctggtacttgttaacccgtctgtatccaccgatgca
gttgcaacggccatagcacaagacgtccacacaacgttggggataacaggacttggggca
gggattgccacaggctacctgcacctcgttcctgccgcagatggaactgccagagatagg
ggaatataggggaagatacagcccaaagatcagtgccagccggaacatggccaacgccaa
cgagtgccggagaagcattgtcgtgggagccctatcaaatggcttttgttctagctgtca
gagcgtggctaaaggtcttattcagttcagtttatacagttctctctccagttggtattt
caaaccatgaa
```

16 dHYSC, MV2-25
Ref Length= 361 bp, Aligned Length= 382 bp, Silent Sites w/o-Gaps= 323 bp

```
>3:17444491,17444852 (reverse complemented)
actagaattttggtgttgacggactgctgctcaacccttggactagttgtaaggcgacct
caaacatatcaagtagctattcttaatggagaaaggattcccaccaaaacactcttagat
cttcagctattgactttcgatgtgtactggtttttgttaccatattgtagactttagaat
cttcgtctgctcgatagaaacactaaggtagttgctattctaggtatactctgctttagg
tgtgtgccatatgttgttctgttgtaggtgcaacgaaagaaatggctaaaatcgatgggc
atttgtgagcaacacttaggaggcttcccttccgctggcagagtgtttctgttatgttca
gc
```

17 dHYST, MV2-25
Ref Length= 896 bp, Aligned Length= 923 bp, Silent Sites w/o-Gaps= 641 bp

```
>3:17705232,17706128
ttattcgaaagtttggattcgttcactcacAATTTCACCCAATAGGACAACATTTTCCCC
TCGGATTATAAACACTCCACGTGGAATGTCACCATATTGATTGCCCACATGTATCCTCTC
AATGGTGCGCTGCAAAACCAAGTTGGCAAACTGATCCACCGATCGTAGGTAGCCAATCAG
GGTCCGACCATCACGCAGGAGCACCATCAGTTTCTctgaaattaagacatttcattgtca
tccaaaaaaacggccggagccactcacTGTCTACTTCTTCCAGGAGGTGAGCTGTGCCCG
CTAAAGGGTTCAGATCGTCCATgtgcagactgtgggtgattgattgtgtgtagaaaaacg
acttcgattcaacttttcgcttaattattgttaaatgtgttttttttttttcgagttctta
agtgctcctgctattcttgagtagcgtgggattatttggccgcccgactatgcgaagcag
gaaattgccaagtcaaaggatggaagttgttggtgtactagttataagtcaattagtgtt
cggcacacgctgatgttttctttttcaaaaaatttataaatatcattatactttcttaga
gatggcagtgcttcgataattcacgcgacagtgatcgcaggtggtggcagttcctcaata
gcacaatacaggaataaaatatcgatgcaaagtatcgattcaccgatatttttgattgtt
gacgttatcgagcagctgagccggcatgcggccaccattagtttgaaaaattgaaaattg
aatcgaaatagcaggaacATGAAgtaaatttgtgattaactgatctaaactgttgctaaa
accattggaaatccaacccttagGATCAGTATTGACGGCTTGCTCGTCTACTTCCCG
```

Coding Information (Note: This region includes two genes on opposite strands. GA25100 is on the plus strand and GA18077 is on the minus strand)
dHYST analysis of (324..923) GA25100 coding regions

Region	Alignment Coordinates	Actual Coordinates
Noncoding	1..819	17705232..17706030
Exon 1 (position 1) GA25100	820..824	17706031..17706035
Intron 1	825..889	17706036..17706095
Exon 2 (position 3) GA25100	890..923	17706096..17706129

dHYSTc analysis of (601..923) GA18077 coding regions

Region	Alignment Coordinates	Actual Coordinates
Noncoding	1..600	17706129..17705555
Exon 1 (position 1) GA18077	601..655	17705554..17705550
Intron 1	656..707	17705499..17705448
Exon 2 (position 2) GA18077	708..892	17705447..17705263
Intron 2	893..923	17705262..17705232

18 Ecdysone Receptor, MV2-25
Ref Length= 335 bp, Aligned Length= 343 bp, , Silent Sites w/o-Gaps= 127 bp

```
>3:17896399,17896734 (reverse complemented)
AGCGGCGCGAGAAGAAGGCCCAGAAGGAGAAGGACAAGATGACCACCTCGCCCAGCTCCC
AGCACGGCGGCAATGGCGCCGGGGGTGGTAATAGTGTGGGCGGCGGCTCCAGCCTTGGCG
GCGGCAGCGTCGGTGGTACCCATGACTACGTGAAGAAGGAGATTCTCGATCTGATGACAT
GCGAGGCGCCGCAGCATGCCACAATTCCGgtaagttgcagcaagcggatcgcaccagatt
ccctgactgatctcactctctctctctctctcttctggcagCTACTACCTGATGATATTT
TGGCCAAGTGTCAAGCGCGCAATATACCTCCACTAA
```

Coding Information GA14596

Region	Alignment Coordinates	Actual Coordinates
Exon 2 (position 2)	1..209	17896734..
Intron 2	210..288	
Exon 3 (position 1)	289..343	..17896399

Frequency Spectra of Derived Mutations Within the Five Gene Arrangements. We used the *D. miranda* sequence to polarize the mutations within *D. pseudoobscura* to better understand the accumulation of new variants in the different gene arrangement backgrounds. We observed 758 polymorphic sites in the 18 regions. At 88.3 % of the 758 segregating sites, the most frequent *D. pseudoobscura* nucleotide matches the *D. miranda* nucleotide leading to the inference that the derived nucleotide is generally the lower frequency base. *D. miranda* has an inferred indel mutation at 3.3 % of the *D. pseudoobscura* segregating sites, which prevents an unambiguous inference of the derived base. For these sites, we made the conservative assumption that the majority base is the ancestral base, which will lead to an underestimate of the derived base frequency at the site if our ancestral base inference is wrong. Figure S3 shows the frequency spectra data for each segregating site.

Figure S4 shows the derived frequency spectra for segregating sites at breakpoint and non-breakpoint regions. The major difference between the two frequency spectra is in the relative frequencies of rare and fixed derived mutations. The breakpoint regions have a slightly lower frequency of rare derived variants and a higher frequency of fixed derived mutations than the non-breakpoint regions. In breakpoint regions, the frequency of rare variants increases as the age of the gene arrangement increases while this trend is less obvious in the non-breakpoint regions.

Unique, Shared and Fixed Polymorphisms Among Breakpoint and Non-breakpoint Regions of the *D. pseudoobscura* Gene Arrangements. We examined the distribution of the 758 polymorphisms among the five gene arrangements to determine whether they are unique to a particular arrangement, shared among arrangements, or represent a fixed difference within a gene arrangement. Each polymorphic site was classified into one of six categories based on the distribution of polymorphism among the five gene arrangements. Category 0 polymorphisms occurred when a segregating site was fixed in one to four gene arrangements. Category 1 polymorphisms occurred when a site was polymorphic in one arrangement and monomorphic in the other four arrangements (five possible configurations). Category 2 polymorphisms were defined when a site was polymorphic in two arrangements and monomorphic in the other three arrangements (ten possible combinations). Category 3 polymorphisms were indicated when a site was polymorphic in three arrangements and monomorphic in the other two arrangements (ten possible combinations). Category 4 polymorphisms occurred when a site was polymorphic in all, but one arrangement (five possible configurations). Finally, Category 5 polymorphisms were defined by sites that were polymorphic in all five arrangements.

The observed numbers of the six categories and 32 possible configurations of polymorphisms are shown in Table S7. We asked whether the observed frequencies of the polymorphism configurations depart from expectations derived from the observed polymorphism frequencies within the five arrangements. We used the fraction of sites that were polymorphic in each arrangement to estimate the joint probability for each outcome (AR, 302/ 758 = 0.398; PP 248/ 758 =0.327; ST, 218/ 758=

0.288; CH, 321 / 758=0.423; TL, 138/ 758= 0.182). If p is the fraction of polymorphic sites within arrangement, then the monomorphic fraction was obtained from 1- p. A chi-square goodness-of-fit test rejects the hypothesis that the distribution of polymorphisms among the five arrangements is independent (X^2=512.7, df=31, P=3.7 x 10^{-91}). The residuals show that there is an excess of category 1 polymorphisms (466 Observed versus 258.6 Expected), which are sites polymorphic in one arrangement and not in the others. The largest excess of sites occurs within the AR arrangement (140 Observed versus 68.3 Expected). Other configurations with a significant excess of sites are found in the Categories 3, 4, and 5. In category 3, there is a significant excess of sites where the polymorphism is shared among PP, CH, and TL. In category 4, AR, PP, ST, and CH are polymorphic and TL is monomorphic for more sites than expected (44 Observed versus 9.8 Expected). Of the 44 sites in this configuration, 11 sites represent fixations of the derived allele in the TL lineage.

We tested whether the distribution of polymorphic sites into the six categories was homogeneous between breakpoint and non-breakpoint regions with a chi-square test of homogeneity (Table S8). The category distribution is not homogeneous between breakpoint and non-breakpoint regions (X^2=25.4, df=5, P=0.0001). Two categories are responsible for rejecting the null hypothesis of homogeneity, categories 1 and 4 in non-breakpoint regions. Both cases are due to a significant deficiency of sites within non-breakpoint versus breakpoint loci.

We tested whether the frequency of unique polymorphisms within each arrangement was proportional to the frequency of total polymorphisms within each arrangement. We rejected the hypothesis of homogeneity with the AR arrangement having a significant excess of unique polymorphisms (X^2=16.5, df=4, P=0.002) (Table S9).

We observed 25 category 0 polymorphisms where all arrangements are monomorphic for a single nucleotide, but at least one arrangement is fixed for a derived variant. Of these polymorphisms, only one site is found in a non-breakpoint region (Amy1), six polymorphic sites in the dSTAR regions are fixed for the derived mutation only in the AR chromosome, and 11 polymorphic sites in the dHYSC region are fixed for the derived mutation in the TL arrangement. Overall, fixed derived mutations in just the TL chromosome accounts for 14 of the 25 category 0 polymorphic sites, which is greater than expected given the relative proportion of polymorphic sites in TL (X^2=30.5, df=2, P=3.4x10^{-7}) (Table S10).

Overall, this analysis shows that each arrangement has accumulated unique polymorphisms with the AR, one of the youngest arrangements, having the greatest number of unique polymorphisms (Table S7). Breakpoint regions, however, do not have a significant excess of unique polymorphisms compared to non-breakpoint regions (Table S8).

Table S1 Primer Sequences Used for PCR Amplification

Primer Name	Primer Sequence	Coordinate Interval	Length (bp)	Cytological Position	Annealing Temp (° C)
pSTPP_f	GAT ACC ACT CGG CAA GCA GAA G	2,292,752…2,293,105	354	64C	55
pSTPP_r	CGC CTC AGT TAA TTA GCC CAC AAA				
pHYSC_f	TGG TGT TGA GTA TCT GCC GTG GTT	6,432,216…6,432,591	376	68C	60
pHYSC_r	CTG CTG CCG CTG CTC CTA TCA				
pSTAR_f	CCT GAT ACC CAC GGA GTC TTC	8,900,356…8,900,823	468	76B	55
pSTAR_r	TCG CTA CAG GGA TCA GGT TTT				
pHYST_f	CTT ATT CCC GCC TCT TGT GTA GC	9,140,888..9,141,693	806	76B	60
pHYST_r	GAC GGC CCT CAG ACG ATA GTT G				
dSTPP_f	ATC GGT ACA ACA GCC AGG GAC AAC	9,832,256…9,832,828	573	75B	58
dSTPP_r	ACT TCG TGG GAT CGC TGG CAT AAT				
dSCTL_f	ATG GCG ATG GAG TCC TCT GTC TAT	10,830,478...10,830,881	404	74B	60
dSCTL_r	ACT GGC GCC ATG TCT CTG TCT CG				
pSCCH_f	AAC CGG CAT ACA CCC TCA TTC	14,259,606…14,259,984	377	70C	57
pSCCH_r	GTT GCG CAT TAT TTA TTC CCT GTA				
dSCCH_f	TCC GGA GAT CGC AAA ACT GTC G	15426293…15426646	379	77B	55
dSCCH_r	TAT GCG CTG CTT CTG ATG CTT GAT				
dHYSC_f	GAG CCC GGG CCA GGT CCA T	17,444,491…17,444,852	362	79C	58
dHYSC_r	TAT CGT GCG TTG TGC GTA ATC AGC				

dHYST_f	ACA AGA TCC GGG GTA TTA	17,705,2323..17,706,128	898	79D/80A
dHYST_r	CTG TTC CGG GTA GAT GTA TTC GTA			

The abbreviated name represents the location (p, proximal or d, distal) of the breakpoint on the chromosome, and the last four letters represent the two arrangements involved in the inversion. The first two letters are the ancestral arrangement and the last two letters are the derived arrangement. For example, the STPP notation is for the breakpoints that converted the ancestral Standard arrangement into the derived Pikes Peak arrangement. Primer names are the abbreviated region names with the addition of “f” and “r” for forward or reverse. The coordinates are the location of the genetic marker in the genome strain (RICHARDS *et al.* 2005), which carries the Arrowhead arrangement.

Table S2 Nucleotide Polymorphism in 18 Regions of the *D. pseudoobscura* Third Chromosome.

Region **(Silent Sites)**	***Sample***	***n***	***S***	***θ ± Sd***	***k***	***π ± Sd***	***Tajima's D***
**pSTPP (353 bp)**	All	91	26	0.014 ± 0.004	3.519	0.010 ± 0.005	-0.945
	AR	23	14	0.011 ± 0.004	2.842	0.008 ± 0.004	-0.887
	PP	20	12	0.010 ± 0.004	3.321	0.009 ± 0.005	-0.065
	ST	21	11	0.009 ± 0.004	2.919	0.008 ± 0.005	-0.159
	CH	22	11	0.009 ± 0.004	3.108	0.009 ± 0.005	0.104
	TL	4	2	0.003 ± 0.002	1.000	0.003 ± 0.002	-0.710*
en **(302 bp)**	All	142	46	0.029 ± 0.008	3.603	0.012 ± 0.006	-1.728*
	AR	50	27	0.020 ± 0.007	2.830	0.009 ± 0.005	-1.748*
	PP	25	8	0.007 ± 0.003	1.740	0.006 ± 0.003	-0.567
	ST	27	18	0.015 ± 0.006	2.621	0.009 ± 0.005	-1.544
	CH	32	15	0.012 ± 0.005	4.763	0.016 ± 0.008	0.927
	TL	7	9	0.012 ± 0.007	3.428	0.011 ± 0.007	-0.355
**pHYSC/pSCTL (193 bp)**	All	93	27	0.027 ± 0.008	5.200	0.027 ± 0.013	-0.051
	AR	23	6	0.008 ± 0.004	0.885	0.005 ± 0.003	-1.394
	PP	19	15	0.022 ± 0.009	4.971	0.026 ± 0.013	0.592
	ST	23	11	0.015 ± 0.007	1.375	0.007 ± 0.005	-1.843*
	CH	23	12	0.017 ± 0.007	2.300	0.012 ± 0.007	-1.014
	TL	4	11	0.031 ± 0.018	5.500	0.028 ± 0.017	-0.837
exu 1 **(176 bp)**	All	144	17	0.018 ± 0.006	1.312	0.007 ± 0.005	-1.546
	AR	53	8	0.010 ± 0.004	1.066	0.006 ± 0.004	-1.067
	PP	26	3	0.004 ± 0.003	0.671	0.004 ± 0.003	-0.359
	ST	25	5	0.008 ± 0.004	1.319	0.008 ± 0.005	-0.011

	CH	32	5	0.007 ± 0.004	1.402	0.008 ± 0.005	0.347
	TL	7	2	0.005 ± 0.004	0.762	0.004 ± 0.004	-0.274
**pSTAR (440 bp)**	All	90	83	0.037 ± 0.010	9.559	0.022 ± 0.010	-1.374
	AR	24	35	0.021 ± 0.008	8.221	0.019 ± 0.009	-0.468
	PP	19	31	0.020 ± 0.008	8.795	0.020 ± 0.010	-0.033
	ST	22	45	0.028 ± 0.010	9.918	0.023 ± 0.011	-0.772
	CH	20	41	0.026 ± 0.010	10.147	0.023 ± 0.011	-0.487
	TL	4	10	0.012 ± 0.007	5.167	0.012 ± 0.007	-0.527
**pHYST 484 bp)**	All	90	59	0.024 ± 0.007	8.854	0.018 ± 0.009	-0.776
	AR	23	29	0.016 ± 0.006	7.154	0.015 ± 0.007	-0.340
	PP	19	33	0.019 ± 0.007	10.443	0.022 ± 0.010	0.423
	ST	19	10	0.006 ± 0.003	3.846	0.008 ± 0.004	1.221
	CH	24	23	0.013 ± 0.005	5.594	0.012 ± 0.006	-0.339
	TL	4	21	0.024 ± 0.013	10.501	0.022 ± 0.013	-0.854
**dSTPP (523 bp)**	All	90	43	0.016 ± 0.005	7.918	0.015 ± 0.007	-0.210
	AR	24	21	0.011 ± 0.004	3.896	0.007 ± 0.004	-1.125
	PP	19	4	0.002 ± 0.001	0.926	0.002 ± 0.001	-0.562
	ST	19	7	0.004 ± 0.002	1.109	0.002 ± 0.001	-1.490
	CH	22	22	0.012 ± 0.004	5.104	0.010 ± 0.005	-0.578
	TL	4	0	0.000 ± 0.000	0.000	0.000 ± 0.000	
**dSCTL (374 bp)**	All	92	47	0.025 ± 0.007	8.242	0.022 ± 0.010	-0.341
	AR	22	11	0.008 ± 0.004	2.649	0.007 ± 0.004	-0.423
	PP	19	17	0.013 ± 0.005	4.749	0.013 ± 0.006	-0.090
	ST	22	9	0.007 ± 0.003	2.009	0.005 ± 0.003	-0.624
	CH	23	22	0.016 ± 0.006	4.893	0.013 ± 0.007	-0.665

	TL	4	8	0.012 ± 0.007	5.000	0.013 ± 0.008	1.442
***eve* (146 bp)**	All	142	24	0.031 ± 0.010	3.337	0.023 ± 0.012	-0.659
	AR	51	6	0.009 ± 0.004	1.471	0.010 ± 0.006	0.261
	PP	26	10	0.018 ± 0.008	3.441	0.024 ± 0.012	1.025
	ST	26	2	0.004 ± 0.003	0.224	0.002 ± 0.002	-1.226
	CH	31	17	0.029 ± 0.011	3.015	0.021 ± 0.011	-0.991
	TL	7	0	0.000 ± 0.000	0.000	0.000 ± 0.000	
***Mef 2* (315 bp)**	All	125	43	0.026 ± 0.007	5.122	0.016 ± 0.008	-1.095
	AR	40	10	0.007 ± 0.003	1.654	0.005 ± 0.003	-0.884
	PP	26	12	0.010 ± 0.004	2.718	0.009 ± 0.005	-0.457
	ST	24	10	0.009 ± 0.004	1.166	0.004 ± 0.002	-1.884*
	CH	27	17	0.014 ± 0.005	3.534	0.011 ± 0.006	-0.695
	TL	7	12	0.016 ± 0.008	3.808	0.012 ± 0.007	-1.212
***Amy 1* (363 bp)**	All	142	51	0.027 ± 0.007	5.369	0.015 ± 0.007	-1.284
	AR	50	20	0.012 ± 0.004	3.082	0.008 ± 0.004	-0.988
	PP	25	3	0.002 ± 0.001	0.661	0.002 ± 0.001	-0.417
	ST	27	13	0.009 ± 0.004	2.323	0.006 ± 0.004	-1.051
	CH	32	11	0.008 ± 0.003	1.942	0.005 ± 0.003	-0.917
	TL	7	13	0.015 ± 0.007	5.046	0.014 ± 0.008	-0.268
**pSCCH (326 bp)**	All	86	29	0.018 ± 0.005	5.026	0.015 ± 0.008	-0.398
	AR	21	7	0.006 ± 0.003	0.981	0.003 ± 0.002	-1.607
	PP	20	8	0.007 ± 0.003	3.747	0.011 ± 0.006	2.230*
	ST	21	5	0.004 ± 0.002	0.476	0.001 ± 0.001	-1.982*
	CH	19	8	0.007 ± 0.003	3.275	0.010 ± 0.005	1.474
	TL	4	0	0.000 ± 0.000	0.000	0.000 ± 0.000	

**dSTAR(*vg*) (309 bp)**	All	143	95	0.058 ± 0.015	17.799	0.058 ± 0.026	0.119
	AR	52	20	0.014 ± 0.005	3.319	0.011 ± 0.006	-0.793
	PP	23	27	0.024 ± 0.009	7.858	0.025 ± 0.012	0.280
	ST	28	25	0.021 ± 0.008	7.818	0.025 ± 0.012	0.782
	CH	31	43	0.035 ± 0.012	5.040	0.016 ± 0.008	-1.956*
	TL	7	33	0.044 ± 0.021	11.097	0.036 ± 0.019	-1.008
**dSCCH (85 bp)**	All	90	8	0.019 ± 0.008	1.417	0.017 ± 0.010	-0.251
	AR	22	4	0.013 ± 0.007	1.389	0.016 ± 0.010	0.747
	PP	20	1	0.003 ± 0.003	0.101	0.001 ± 0.002	-1.158
	ST	22	1	0.003 ± 0.003	0.369	0.004 ± 0.004	0.600
	CH	21	2	0.007 ± 0.005	0.560	0.007 ± 0.006	0.017
	TL	4	2	0.013 ± 0.010	1.338	0.016 ± 0.012	1.929*
**F6 (428 bp)**	All	126	57	0.025 ± 0.007	6.463	0.015 ± 0.007	-1.210
	AR	45	31	0.017 ± 0.005	3.663	0.009 ± 0.004	-1.638
	PP	24	24	0.015 ± 0.006	4.598	0.011 ± 0.005	-1.055
	ST	25	16	0.010 ± 0.004	3.367	0.008 ± 0.004	-0.725
	CH	24	22	0.014 ± 0.005	4.533	0.011 ± 0.005	-0.848
	TL	7	5	0.005 ± 0.003	2.571	0.006 ± 0.004	1.287
**dHYSC (323 bp)**	All	87	39	0.024 ± 0.007	4.421	0.014 ± 0.007	-1.359
	AR	24	11	0.009 ± 0.004	3.094	0.010 ± 0.005	0.171
	PP	19	11	0.010 ± 0.004	2.795	0.009 ± 0.005	-0.402
	ST	21	10	0.009 ± 0.004	3.248	0.010 ± 0.005	0.582
	CH	18	12	0.011 ± 0.005	2.497	0.008 ± 0.004	-1.050
	TL	4	7	0.012 ± 0.007	3.500	0.011 ± 0.007	-0.817

**dHYST (641 bp)**	All	94	54	0.016 ± 0.005	8.600	0.013 ± 0.006	-0.596
	AR	24	27	0.011 ± 0.004	9.115	0.014 ± 0.007	0.975
	PP	19	30	0.013 ± 0.005	10.256	0.016 ± 0.008	0.773
	ST	23	18	0.008 ± 0.003	4.593	0.007 ± 0.004	-0.212
	CH	23	36	0.015 ± 0.005	7.125	0.011 ± 0.005	-1.037
	TL	4	0	0.000 ± 0.000	0.000	0.000 ± 0.000	
**EcR (127 bp)**	All	142	21	0.031 ± 0.010	1.532	0.012 ± 0.007	-1.669*
	AR	51	16	0.028 ± 0.010	1.793	0.014 ± 0.008	-1.530
	PP	25	6	0.012 ± 0.006	1.073	0.008 ± 0.006	-0971
	ST	26	4	0.008 ± 0.005	1.323	0.010 ± 0.007	0.700
	CH	32	8	0.016 ± 0.007	1.573	0.012 ± 0.008	-0.623
	TL	7	2	0.006 ± 0.005	0.572	0.004 ± 0.004	-1.234

n, sample size; *S,* number of segregating sites; θ ± sd, nucleotide heterozygosity per site based on the number of segregating sites with its standard deviation (WATTERSON 1975); π ± sd, nucleotide heterozygosity per site based on the number of pairwise differences with its standard deviation assuming no recombination (TAJIMA 1983); Tajima's (1989) *D*, a test of the frequency spectrum's departure from expectations of the neutral theory where values of Tajima's *D* marked with an asterisk have a probability less than 0.05.

Table S3 HKA test for the Arrowhead gene arrangement

Gene	AR_Obs_S	AR_Exp_S	Dmir_Obs_D	AR_Exp_D
pSTPP	14	11.04	5.28	8.25
en	27	18.74	3.28	11.54
pHYSC	6	8.40	8.68	6.28
exu1	8	5.57	0.96	3.39
pSTAR	35	33.29	22.87	24.59
pHYST	29	28.64	21.03	21.40
dSTPP	21	17.70	9.77	13.07
dSCTL	11	14.10	13.77	10.67
eve	6	6.56	4.58	4.02
Mef2	10	13.42	12.12	8.70
Amy1	20	17.74	8.66	10.92
pSCCH	7	11.72	13.71	8.99
dSTAR	20	24.39	19.28	14.89
dSCCH	4	4.42	3.77	3.35
F6	31	26.80	12.70	16.90
dHYSC	11	12.93	11.48	9.55
dHYST	27	35.41	34.56	26.15
EcR	16	12.13	3.57	7.44
T	1.76			
X2	16.17			
P	0.212			
sim	9982			

Table S4 HKA test for the Pikes Peak gene arrangement

Gene	PP_Obs_S	PP_Exp_S	Dmir_Obs_D	PP_Exp_D
pSTPP	12	9.43	5.28	7.85
en	8	6.33	3.28	4.95
pHYSC	15	12.83	8.68	10.84
exu1	3	2.23	0.96	1.73
pSTAR	31	29.2	22.87	24.68
pHYST	33	29.29	21.03	24.75
dSTPP	4	7.46	9.77	6.31
dSCTL	17	16.68	13.77	14.09
eve	10	8.22	4.58	6.36
Mef2	12	13.6	12.12	10.52
Amy1	3	6.54	8.66	5.12
pSCCH	8	11.84	13.71	9.86
dSTAR	27	25.71	19.28	20.57
dSCCH	1	2.6	3.77	2.17
F6	24	20.49	12.70	16.21
dHYSC	11	12.18	11.48	10.3
dHYST	30	34.99	34.56	29.57
EcR	6	5.37	3.57	4.2
T	1.95			
X2	11.67			
P	0.559			
sim	9639			

Table S5 HKA test for the Standard gene arrangement

Gene	ST_Obs_S	ST_Exp_S	Dmir_Obs_D	ST_Exp_D
pSTPP	11	8.22	5.28	8.07
en	18	11.11	3.28	10.18
pHYSC	11	10.05	8.68	9.62
exu1	5	3.08	0.96	2.88
pSTAR	45	34.47	22.87	33.4
pHYST	10	15.44	21.03	15.6
dSTPP	7	8.34	9.77	8.43
dSCTL	9	11.56	13.77	11.2
eve	2	3.41	4.58	3.16
Mef2	10	11.37	12.12	10.75
Amy1	13	11.3	8.66	10.36
pSCCH	5	9.44	13.71	9.27
dSTAR	25	23.31	19.28	20.97
dSCCH	1	2.42	3.77	2.35
F6	16	14.83	12.70	13.87
dHYSC	10	10.84	11.48	10.64
dHYST	18	26.86	34.56	25.7
EcR	4	3.93	3.57	3.64
T	2.53			
X2	18.22			
P	0.145			
sim	9813			

Table S6   HKA test for the Chiracahua gene arrangement

Gene	CH_Obs_S	CH_Exp_S	Dmir_Obs_D	CH_Exp_D
pSTPP	11	9.81	5.28	6.47
en	15	11.45	3.28	6.83
pHYSC	12	12.52	8.68	8.16
exu1	5	3.73	0.96	2.23
pSTAR	41	38.07	22.87	25.8
pHYST	23	26.79	21.03	17.25
dSTPP	22	19.14	9.77	12.63
dSCTL	22	21.66	13.77	14.11
eve	17	13.47	4.58	8.11
Mef2	17	17.93	12.12	11.19
Amy1	11	12.31	8.66	7.35
pSCCH	8	12.86	13.71	8.85
dSTAR	43	38.88	19.28	23.4
dSCCH	2	3.46	3.77	2.31
F6	22	21.11	12.70	13.59
dHYSC	12	13.82	11.48	9.66
dHYST	36	42.73	34.56	27.83
EcR	8	7.25	3.57	4.33
T	1.4			
X2	8.28			
P	0.783			
sim	9875			

Table S7 Shared, unique, and fixed polymorphisms among the five gene arrangements of *D. pseudoobscura*.

Category	Configuration	Obs	Exp	Residuals
0	P() M(AR, PP, ST, CH, TL)	25	103.1	-7.7
1	P(AR) M(PP, ST, CH, TL)	140	68.3	8.7
1	P(PP) M(AR, ST, CH, TL)	81	50.1	4.4
1	P(ST) M(AR, PP, CH, TL)	68	41.6	4.1
1	P(CH) M(AR, PP, ST, TL)	126	75.7	5.8
1	P(TL) M(AR, PP, ST, CH)	51	22.9	5.9
2	P(AR, PP) M(ST, CH, TL)	14	33.2	-3.3
2	P(AR, ST) M(PP, CH, TL)	12	27.6	-3.0
2	P(AR, CH) M(PP, ST, TL)	30	50.1	-2.8
2	P(AR, TL) M(PP, ST, CH)	4	15.2	-2.9
2	P(PP, ST) M(AR, CH, TL)	5	20.2	-3.4
2	P(PP, CH) M(AR, ST, TL)	13	36.8	-3.9
2	P(PP, TL) M(AR, ST, CH)	10	11.2	-0.3
2	P(ST, CH) M(AR, PP, TL)	23	30.6	-1.4
2	P(ST, TL) M(AR, PP, CH)	5	9.3	-1.4
2	P(CH, TL) M(AR, PP, ST)	6	16.9	-2.6
3	P(AR, PP, ST) M(CH, TL)	13	13.4	-0.1
3	P(AR, PP, CH) M(ST, TL)	11	24.4	-2.7
3	P(AR, PP, TL) M(ST, CH)	5	7.4	-0.9
3	P(AR, ST, CH) M(PP, TL)	8	20.2	-2.7
3	P(AR, ST, TL) M(PP, CH)	1	6.1	-2.1
3	P(AR, CH, TL) M(PP, ST)	2	11.2	-2.7
3	P(PP, ST, CH) M(AR, TL)	7	14.9	-2.0
3	P(PP, ST, TL) M(AR, CH)	3	4.5	-0.7
3	P(PP, CH, TL) M(AR, ST)	16	8.2	2.7
3	P(ST, CH, TL) M(AR, PP)	6	6.8	-0.3
4	P(AR, PP, ST, CH) M(TL)	44	9.8	10.9
4	P(AR, PP, ST, TL) M(CH)	0	3.0	-1.7
4	P(AR, PP, CH, TL) M(ST)	6	5.4	0.2
4	P(AR, ST, CH, TL) M(PP)	3	4.5	-0.7
4	P(PP, ST, CH, TL) M(AR)	11	3.3	4.2
5	P(AR, PP, ST, CH, TL) M()	9	2.2	4.6

Category, polymorphism category; Configuration, P() and M() indicate which arrangements were polymorphic or monomorphic at a site, respectively. Obs, observed numbers of each configuration; Exp, expected number of each configuration assuming that the polymorphisms are distributed independently. The colored boxes in the residuals column indicate deficiencies (blue) or excesses (red) of polymorphic sites for a particular configuration.

Table S8   Observed and expected numbers of polymorphic sites classified into the six unique, shared, and fixed categories for the five gene arrangements of *D. pseudoobscura*.

Category	Breakpoint	Non-Breakpoint
0	24 (16.8)	1 (8.2)
1	298 (312.9)	168 (153.1)
2	80 (81.9)	42 (40.1)
3	44 (48.3)	28 (23.7)
4	56 (43.0)	8 (21.0)
5	7 (6.0)	2 (3.0)

Observed (Expected); The blue shaded box indicates a significant deficiency of observed values based on the residuals.

Table S9 Observed and (expected) numbers of unique polymorphic sites for the five gene arrangements of *D. pseudoobscura*.

Arrangement	Unique Polymorphisms	Non-Unique Polymorphisms
CH	126 (121.9)	195 (199.1)
AR	140 (114.7)	162 (187.3)
PP	81 (153.8)	167 (153.8)
ST	68 (82.8)	150 (135.2)
TL	51 (52.4)	87 (85.6)

Table S10 Observed and (expected) numbers of category 0 polymorphic sites for the three gene arrangements of *D. pseudoobscura*.

Arrangement	Unique Fixed Polymorphisms	Non-Unique Fixed Polymorphisms
AR	6 (9.2)	296 (292.8)
PP	1 (7.6)	247 (240.4)
TL	14 (4.2)	124 (133.8)

File S2

Literature Cited

RICHARDS, S., Y. LIU, B. R. BETTENCOURT, P. HRADECKY, S. LETOVSKY *et al.*, 2005 Comparative genome sequencing of *Drosophila pseudoobscura*: Chromosomal, gene and *cis*-element evolution. Genome Research **15:** 1-18.

TAJIMA, F., 1983 Evolutionary relationship of DNA sequences in finite populations. Genetics **105:** 437-460.

TAJIMA, F., 1989 Statistical method for testing the neutral mutation hypothesis by DNA polymorphism. Genetics **123:** 585-595.

WATTERSON, G. A., 1975 On the number of segregating sites in genetical models without recombination. Theoretical Population Biology **7:** 256-276.
