# Supplementary material for: Molecular Population Genetics of Inversion Breakpoint Regions in Drosophila pseudoobscura
Source: G3 (Bethesda). 2013 Jul 1;3(7):1151–63. doi: 10.1534/g3.113.006122 (PMC3704243; doi:10.1534/g3.113.006122)
Supplement: Supporting Information [file supp_g3.113.006122_FigureS1.pdf]

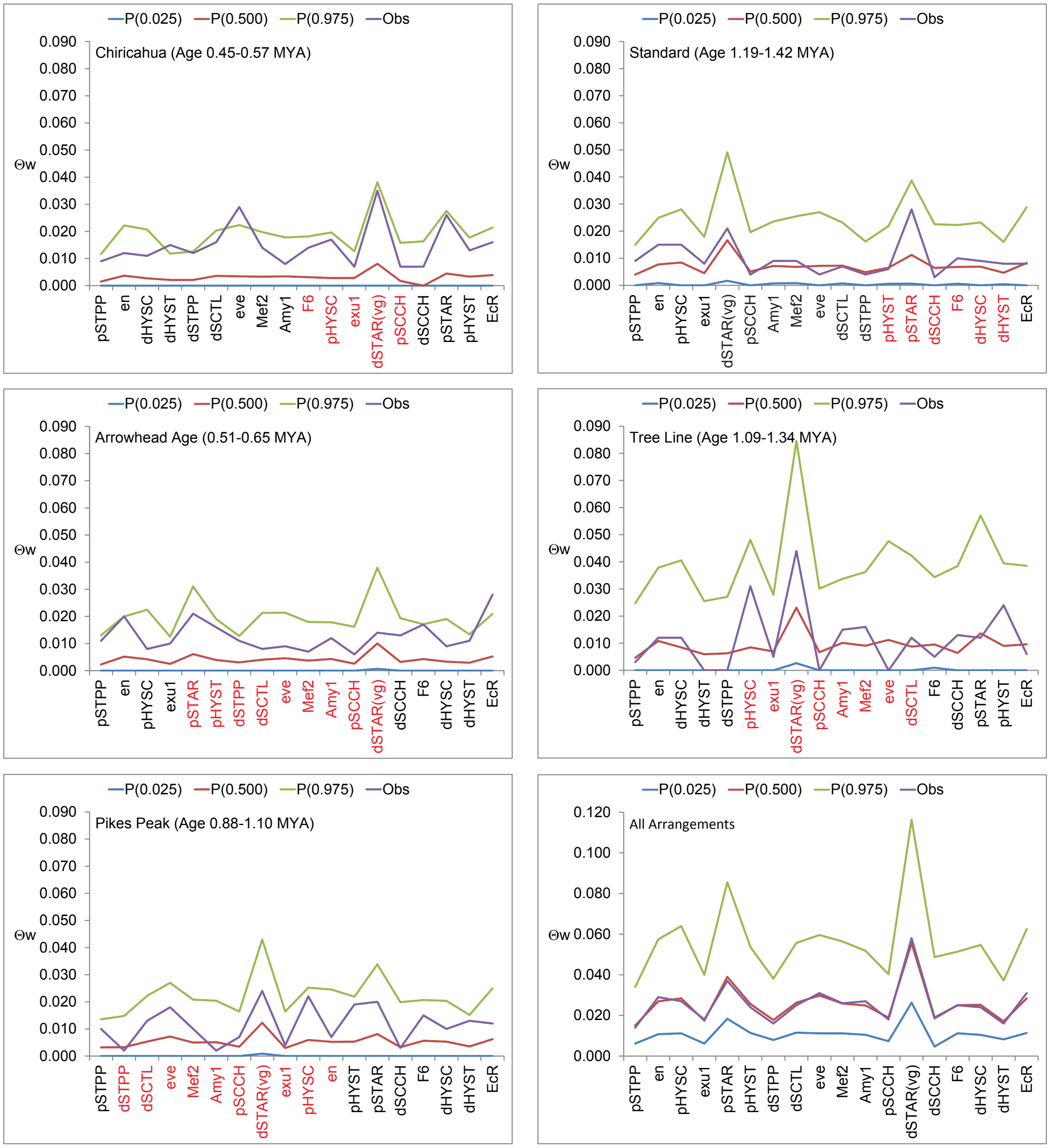

Figure S1 Observed versus expected estimates of nucleotide heterozygosity (Θw) at 18 *D. pseudoobscura* marker loci based on a coalescent analysis of a population of constant size. Nucleotide heterozygosity estimates based on the number of segregating sites (Θw) within five gene arrangements (AR, PP, CH, ST, and TL) as well as among all arrangements are shown in the individual panels. The observed (Obs) estimates of Θw as well as the mean P(0.500) and the 95% confidence interval (P(0.025) to P(0.975)) derived from 1000 coalescent simulations using nested subsamples for a constant population size model. The genetic markers labeled in red on the x-axis are genes within the inverted region of the derived arrangement. The proximal region is to the left.
