# Supplementary material for: Molecular Population Genetics of Inversion Breakpoint Regions in Drosophila pseudoobscura
Source: G3 (Bethesda). 2013 Jul 1;3(7):1151–63. doi: 10.1534/g3.113.006122 (PMC3704243; doi:10.1534/g3.113.006122)
Supplement: Supporting Information [file supp_g3.113.006122_FigureS3.pdf]

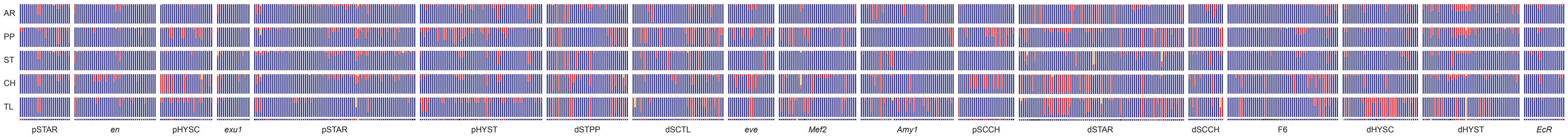

Figure S3 Frequency of variants at the segregating sites in the five gene arrangements. The five bars represent the frequencies of variants in the five gene arrangements. Each column represents a single segregating site. For each site, the colors of segregating bases from most frequent to least frequent in decreasing order are: blue, red, orange, and purple. The bottom line indicates the base of *D. miranda*, where an indel is designated by a black square. The segregating sites of the different gene regions are separated by white space.
