# Supplementary material for: Molecular Population Genetics of Inversion Breakpoint Regions in Drosophila pseudoobscura
Source: G3 (Bethesda). 2013 Jul 1;3(7):1151–63. doi: 10.1534/g3.113.006122 (PMC3704243; doi:10.1534/g3.113.006122)
Supplement: Supporting Information [file supp_g3.113.006122_FigureS4.pdf]

Breakpoint Regions

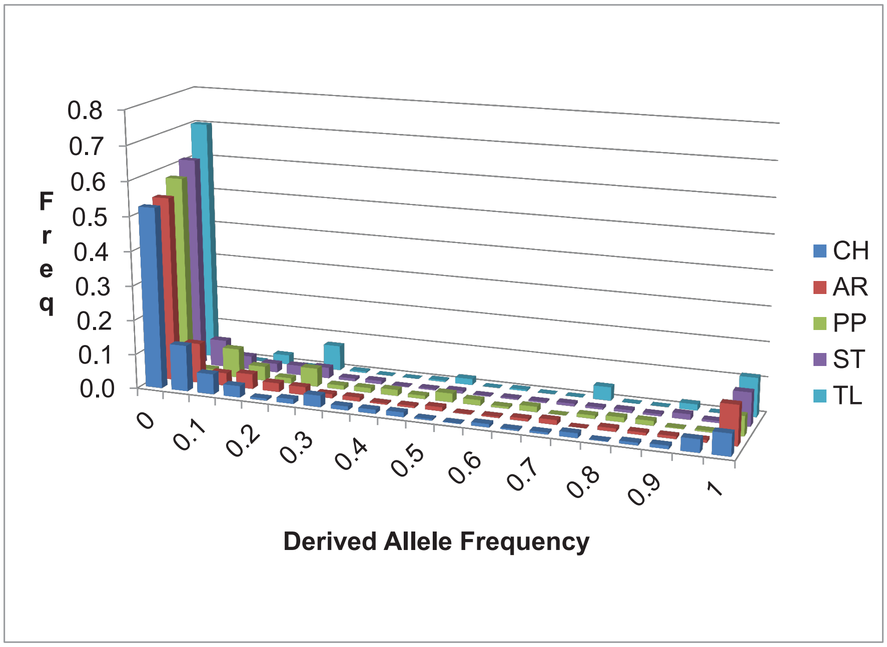

Non-Breakpoint Regions

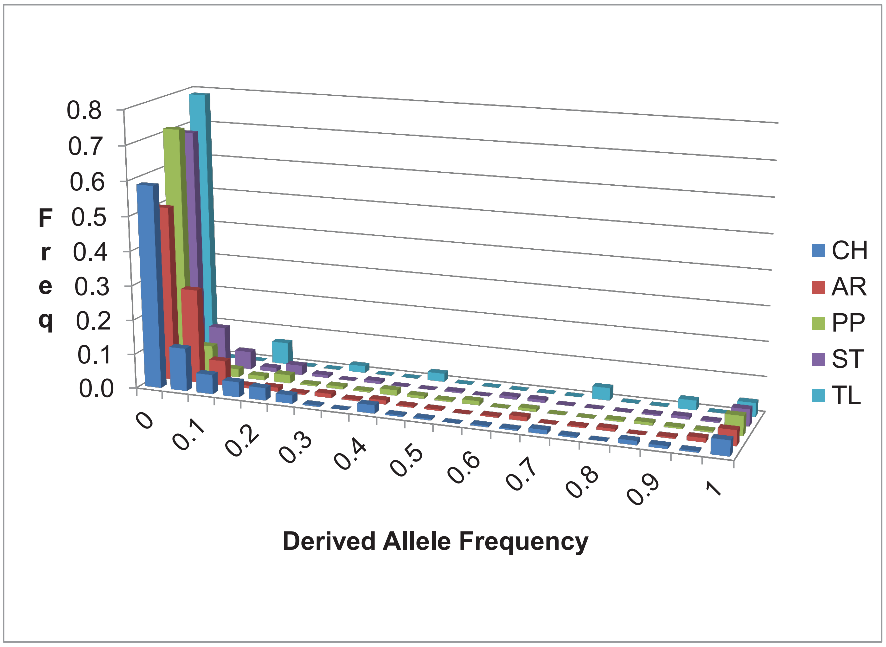

Figure S4 Frequency spectra of derived mutations in breakpoint and non-breakpoint regions for five gene arrangements of *Drosophila pseudoobscura*. A total of 509 and 249 segregating sites were in breakpoint and non-breakpoint regions, respectively. The relative frequency of sites is displayed on the z-axis, which is the number of sites that have a particular site frequency divided by the total number of sites. This allows the frequency spectra of the two types of regions to be compared. The arrangements are ordered on the y-axis from youngest (CH in front) to oldest (TL in back).
