# Supplementary material for: Molecular Population Genetics of Inversion Breakpoint Regions in Drosophila pseudoobscura
Source: G3 (Bethesda). 2013 Jul 1;3(7):1151–63. doi: 10.1534/g3.113.006122 (PMC3704243; doi:10.1534/g3.113.006122)
Supplement: Supporting Information [file supp_g3.113.006122_FileS1.pdf]

File S1

Supporting Data

***D. pseudoobscura* Inversion Gene Markers**

The following list provides the sequences of the 18 gene markers in the *D. pseudoobscura* reference strains MV2-25; (Richards *et al.* 2005) used in this study. The title shown in bold gives the number of the marker, the name of the marker, the reference genome strain, the length of the amplified region excluding primers in the reference genome, the length of the aligned sequence. The coordinates for the FASTA entry are given on second line based on release (R2.27) in FlyBase (http://flybase.org) where 3 indicates chromosome 3 of *D. pseudoobscura*. The noncoding sequence is shown in lower case letters and coding sequence is indicated in upper case letters. Coding information is shown below the sequence.

01 pSTPP, MV2-25
Ref Length=354 bp, Aligned Length= 363 bp, Silent Sites w/o-Gaps = 353 bp
>3:2292752,2293105
gacgaaaccaaaacctaaacagaactcattttgattcacataacagtgggctgtccgcgg
tctggaaaccatcatttagcatcaaacgtaccaataaaatataataataaccaaacttat
caaagcgaatgttaattagtgcggaaattcacactaatctgcgtgcttccaggctcgttt
ccatgtaattcgtttatccatcagtgccatctatctattccgccgcaaatgggcatttaa
ttaatgtccaaattcgattttagacaaatgtttatccatcacatcaatacaaatctaaat
tgtgcgtgtttgcggtatcctcgcacttgccagcacattttacctttgacgtta

02 engrailed, MV2-25
Ref Length=376 bp, Aligned Length= 407 bp, , Silent Sites w/o Gaps = 302 bp
>3:4595297,4595673 (reverse complemented)
cacacaagcactcacacagacccatgtagaagccatagatcccttgttgtgtcatatttg
caccaaaatatttacacatattttccgagaccccgcggcgattgcatcctggcaaacgc
atgtcctgcctcggtccccggtcccctgtccccggtccgtacttaaccaattagacacgc
tcatatctcacccggacttccatctactggcagtctgtcttcggggtgcctctttcaact
caaaccgcaaataattgatttgccttacctacctaccagcctcccatagtagatcccatc
cgtatctctgtgcctctgtactcatcccagactcctctctgatctttctacttgcagCGG
GAGTTCAACGAGAATCG

Coding information GA21479

Region	Alignment Coordinates	Actual Coordinates
Intron 1	1..386	4595645..4955258
Exon 2 (position 1)	387..407	4955257..4595297

03 pHYSC, MV2-25
Ref Length=375 bp, Aligned Length= 398 bp, , Silent Sites w/o Gaps = 193 bp
>3:6432216,6432591
tatttggacgcagactcagtttaattttcacattttagcccaaaattcactcctagggcc
ccgggcagacaaaggcagacaaactgcgctgcaaccgaataaaccatttacaaatacgag
tagcattcgagcaaatataggcgattggcattctatttgaatgagctgcatggagcgtcg
gtcttaagcttaaggcaattgtttgagtgccttttgctgccgttgatggataggattcaa
atagccagaggcgtgggatcacgtaatgcacttccccagcaaccactgggccacaaaaac
gaggcagcaaataaggcagtggccaaagtcagccactaattgcaacattaaagctccttt
tggttgccactgtgtg
04 exuperantia 1, MV2-25
Ref Length=356 bp, Aligned Length= 358 bp, Silent Sites w/o-Gaps= 176 bp
>3:8591272,8591628
gggcattgaccctctgcgtcggacctaacctaacttttctctacgtctcccctttgccca
ttttccagATTGTCCAGTTGGCTGCCTACACTCCAAAGGACAACTTCCAGCAGTACATCA
TGCCGTATATGAATCTGAATCCAGCCGCTCGTCAGCGTCATCAAATTCGTGTGATTTCGA
TCGGCTTTTATCGCATGCTGAAGTCGATGCAGACCTACAAGgtacattcatacctacact
cccgttatgtccgctaattctagcttttattttctcagATTATCAAATCTAAATCGGAGG
TTGCTGCTCTCATGGACTTTCTCAACTGGCTTGAGATGCTGGTCGCCAAACAGCCCA

Coding Information GA21461

Region	Alignment Coordinates	Actual Coordinates
Intron 1	1.. 68	8591272..8591339
Exon 2 (position 1)	69..221	8591340..8591492
Intron 2	222..279	8591493..8591550
Exon 3 (position 1)	280..358	8591551..8591628

05 pSTAR, MV2-25
Ref Length= 467 bp, Aligned Length= 473 bp, Silent Sites w/o-Gaps= 440 bp

```
>3:8900356,8900823
gatgacaaacatattcttaattctataacatccattcccccagggtatgtgtgcgtggaa
tgggactcattgttgtgaaccggttatgacgactaattcttggttaatctttcttccgtc
gaatattcccagctatatagatctcttatttttgcccagataatttaatatgatagctgt
atcaattttgatcaagattaactatttttaaatactcgcatgtttttttgttttgacaaaa
acacgatttcatcaaaaatgttcaatgccgcaggttgtgacgtcaatgttgctggtattt
gcagattcttttgttgtcaaccagggtatgagcgtttgtataccctatttcgtgaatatt
atataaagatactgttttccaagctgctttcaaatgtaattaataaaggccttgttttag
attgcattttttttggtctattcatgcatttgattagttatttgtatat
```

06 pHYST, MV2-25
Ref Length= 805 bp, Aligned Length= 1,486 bp, Silent Sites w/o-Gaps= 484 bp

```
>3:9140888,9141693
CGCAATGCAAAGCCATGTCGATATTGAAGAATTCTTTATGAAAGTGGATTCAAGTGGTGC
GCACGATCAGACGGTGCACGgtaagtttatagcatatacatttgtgtatattcgctcata
tgctttcttttgatagGTCTTGGCGTATACGATTTGCACGAAATGGTGGAAAAGGCTAA
GAAGATAGATCAACAAATGCAGTCCTCTCAGCCCACCTTTATCCATCATCGCTTTCGCTA
Actgcctttgaaaaactttaagactataacaacaacaaaattattattattcatgtcatt
atttttggatatgcttattgccttactttgttcgattgttcggttatttggaaaaatttc
aatattgtttattgcttctgtcccgggtaccctgtgattatctatttacgattcagtggc
acttcgataagaagccacaaaagcgaaattaaagcaagaatggattgtgcagaagatcgg
atatctttataactatagtttattagtgttcaatcagcgtttagagtaattaaagcaatt
taactctaccatgcataagtgaacacagcctaaaataacacgttcgtgtcattcaaattt
tattgtgctctcaagagcaggaacaatgcgtggccagctgattatttctgtgcttctcgg
attattcctaacattccacatcaatataaacgtaaatataatactagtaaagtggacaat
attgaaccaaatttttagGATGCCGTCGTCTTTAAATTTACAAACGTCGTCTGCGGGAGT
TACAACCAATCCTGGTTCGTGTTCCA
```

Coding Information GA21596 (Dmel CG9183) and GA24829

Region	Alignment Coordinates	Actual Coordinates
Exon 1 (position 3)	1.. 80	9140888.. 9140967
Intron	81.. 145	9140968.. 9141024
Exon 2 (position 2)	146.. 247	9141025.. 9141128
Noncoding	248..1418	9141129.. 9141626
Exon 1 (position 1)	1419..1486	9141627.. 9141693

07 dSTPP, MV2-25
Ref Length= 572 bp, Aligned Length= 587 bp, Silent Sites w/o-Gaps= 523 bp

```
>3:9832256,9832828
GTGGAAAGGGTCCTCAAGTAGactctattctactgaaattttacagctttccaggataaa
aaacgatgtagtttttgttctttttctgactttgttttatacaaaaatgcatctttattc
gtttaattattaaatatacacaagaaatctcatatgttcgtttcttttgcatgttttcca
ttttctcatctcgttttgggatttcctcagctaaaaattaaatacaatgcttatttatat
gaatgtgtgaatgcgttaatggtcatagatgtgatcgagattgtgtgcggtgtgtgacgg
tgtgtgcgtggactaacacaattgaggcgcttaaacaaaacaaacggcaaaaaatacaaa
atggagcacgtggcatagagacagtggtggagataacagaacagctggtagggaggaacg
gagcgcgcctggctatggcaaaaatggcgggcgctccactagagggggatagacataga
gacgagcgggatagagatagatggagtaagacagtcactgtcaaactgttgaggaaccgc
tgcccctggcatcggacttaatactcggccaggg
```

Coding Information GA24854

Region	Alignment Coordinates	Actual Coordinates
Exon (position 1)	1.. 18	9832255..9832275
Noncoding	19..587	9832276..9832828

08 dSCTL, MV2-25

Ref Length= 403 bp, Aligned Length= 444 bp, Silent Sites w/o-Gaps= 374 bp

```
>3:10830478,10830881
gcgtgccaaagtgccaatggcgttttaatttgaagctggagttcatgtcggcgtcagctg
ccggcacagactgggtatcgtttatgagctgccgactgctgttgacttttatttatttaa
tgtttcggctttagtgttttgttttttttgctttttataattttatttggcttttcctgcc
ttggacagagcagagtacgaacctgttcccgacctggctacctgcaaaaacagcaccgag
tggaagatcgatgcaaaactggattaagtcccttttacgccctaatccgcactggacctg
gccctagccctggactcgggtcatcgtgtaatggctaaaagagtgagaaagagaggccga
gtggtagtacgagcgagagagagagagagagcaggcgaatcctgcc
```

09 even-skipped

MV2-25, Ref Length= 373 bp, Aligned Length= 374 bp, Silent Sites w/o-Gaps= 146 bp

```
>3:10904241,10904614 (reverse complemented)
AATCACCATCACGACTCCAATGCCGTGGACCAGAAGCCCCTCGTGGTCGATCTCATGGCC
ACGCAATACGGCAAACCCCAGACGCCGCCGCCTTCTCCAAATGgtaggtttccgcaaagc
ccaatggacacattcgacagattattaacccagcctttaccctcttcttgcagAGTGCCT
CTCCAGTCCGGACAACTCGCTGAACGGCAGCCGCAACTCCGAGATTCCCGCTGATCCGTC
GGTTCGACGCTACCGAACCGCCTTCACACGCGATCAGCTTGGACGTCTCGAGAAGGAGTT
CTACAAGGAAAACTACGTGTCCCGCCCGCGTCGCTGCGAGCTTGCCGCCCAGCTCAACCT
GCCGGAATCCACCA
```

Coding Information GA15349

Region	Alignment Coordinates	Actual Coordinates
Exon 1 (position 1)	1..103	10904614..10904512
Intron 1	104..173	10904511..10904442
Exon 2 (position 2)	174..374	10904441..10904241

10 Myocyte enhancing factor 2
MV2-25, Ref Length= 384 bp, Aligned Length= 413 bp, Silent Sites w/o-Gaps= 315 bp

```
>3:10967159,10967543
CAGGTGGGATGTCCTTGATAAgttagtacagtgtctctggcagaagggtgcacacacatt
tttctatctatcagttgttattgttatcgttatcgttatcgttctcgttctcgttcgatt
tttcgttttattttattcttttgcatgatttcccactgccatccccgcccccacccttaa
aaacttctaacagagaatgtgttttgtgcttatttttgtttgtttttgatttccctcgac
tcacccgccatgaatctaacaccagagttccacaattccaatatacatacatatatagtg
cttcgatctaaggtctaaacgtatctgtgcattttcttatcttcttatcgattggtgcca
tcagTATATCCATCGGGTTCGATGC
```

Coding Information GA12881

Region	Alignment Coordinates	Actual Coordinates
Exon 3 (position 2)	1.. 21	10904614..10904512
Intron 3	22..392	10904511..10904442
Exon 4 (position 2)	393..413	10904441..10904241

11 Amylase 1, MV2-25
Ref Length= 422 bp, Aligned Length= 453 bp, Silent Sites w/o-Gaps= 326 bp

```
>3:11849147,11849569 (reverse complemented)
CTCCGCTGACGATGGTGTCCTGGCCATTCATGTCAACGCCATGTTGTAAggaactcagta
tgtgagaccccaacaaccaatcgagattattactattaaatacacaatgatattatgaaa
tgctcgttgactctgagttgggactgctggaaaggataaagggttagggcaggtgcctgg
gcgtttccagatggtcagtgatatccgtattgtatggcgccaagttgtagagaagcctcc
attcaatccggaagggacgagcagtggggaacgtggaaattaattaaatgctggacaaca
aaggaaagagatgggtcactgcagataagaggcggtggcagatgccaactacaggtgaac
tgaggtggctgtacaacgtgaatagtacgagcgtcgtgtatcgtgcgtcaaacaaggtcc
ccc
```

Coding Information GA24265

Region	Alignment Coordinates	Actual Coordinates
Exon 2 (position 3)	1.. 46	11849569..11849524
Noncoding	47..453	11849520..11849147

12 pSCCH, MV2-25
Ref Length= 378 bp, Aligned Length= 402 bp, Silent Sites w/o-Gaps= 326 bp

```
>3:14259606,14259984
cattccaactgcaacacagcattggtccattagccccaatttgctgggaatgtgggagct
aatgggagtggcggtggccgtacgtgaggagaggcaggcaggcgagcaaagagctcgggg
ttattgccacaggttgttgctggccagagggagagcacaattataatttttttaatctaaa
cccgatgacatgatcgaaaaactactctttttacggcaattaaaattgtaatctaaatat
gatgataaataatgatacaaaaaaatgactagagcattgtaaatgttcattagtgcgtttt
atatatccaaactaatacttaagtcccaagagtatttgtttttcaaaaatatctgcctga
ccctgctcttctctttagc
```

13 dSTAR, MV2-25

Ref Length= 516 bp, Aligned Length= 651 bp, Silent Sites w/o-Gaps= 309 bp

```
>3:14801829,14802345
gagacacgcattgtttctctctctctctgtcgatctctgtctgtggcttgtttacattag
tatctggctgaaattgccggtatagtccagataacagtattcataattcttataagatcg
ttgagaaaacttatctagtagccccagcagcacaaaactttcttctattacgctggaatt
tcaaaaatttcatttgatgttggtactttagtggaggcctgcaagccaatatctagatgt
cgatatatgtatatatcatagcagtagtttttggcaacgtcactcatagaaagagagaga
gaaagcaccagtggataccatttcacactcgcgctaattactttacaaattctttgaatt
gcggacgggcactcgcattccaaaccagttaacattcaataaatgatatcaaattttata
aacccgtacccgtaatatccgtactgattgtgaaatatttctccgatttcgctttttagC
ACGTTTGGTGGGCTCCCAACAGCACCAGCATCACAAC
```

Coding Information GA17716

Region	Alignment Coordinates	Actual Coordinates
Intron	1..613	14801829..14802227
Exon (position 2)	614..651	14802228..14802345

14 dSCCH, MV2-25

Ref Length= 353 bp, Aligned Length= 354 bp, Silent Sites w/o-Gaps= 85 bp

```
>3:15426293,15426646 (reverse complemented)
CATCAGTTCCAGGCGGTGCTCCTGGCCCTCAGGACTCTCCAAAGGCGCAAAGGTGGAAAA
CAGAAGGCGAAGGATCTTCGCCGCATAGCAGCCCTGCATTTGGACACATATCACCTGGCC
AGGCACTTCTTTGGCCTGTACGATGTTGCCAATGCAATGTTGTTTATCAATATGTGTGTG
ACCACTACTAGCATCCTGTACCATGCCGTGCAGTACAGGAACCAGTCAATCCCATCCGAC
GGCTGGGGTAACCTCTTTGGCAGTGGCCTTGTTTTGTTCAACTTGTGCGGGACTCTGATG
CTCATGGAAAAGCTGGATCGAGTGGTCAGCTCGTGCAATGTGGGCCCGGCCCTT
```

Coding Information Ga12328

Region	Alignment Coordinates	Actual Coordinates
Exon 1 (position 1)	1..354	15426293..15426646

15 F6, MV2-25

Ref Length= 430 bp, Aligned Length= 434 bp, Silent Sites w/o-Gaps= 428 bp

>3:16605742,16606172 (reverse complemented)
TTCATggtttgaaataccaactggagagagaactgtataaactgaactgaataagacctt
tagccacgctctgacagctagaacaaaagccatttgatagggctcccacgacaATGCTTC
TCCGGCACTCGTTGGCGTTGGCCATGTTCCGGCTGGCACTGATCTTTGGGCTGTATCTTC
CCCTATATTCCCCTATCTCTGGCAGTTCCATCTGCGGCAGGAACGAGGTGCAGGTAGCCT
GTGGCAATCCCTGCCCCAAGTCCTGTTATCCCCAACGTTGTGTGGACGTCTTGTGCTATG
GCCGTTGCAACTGCATCGGTGGATACAGACGGGTTAACAAGTACCAGGGGCCCTGTGTCC
TTCCCAGCGAGTGTGGAAGGTATCCCATGTTGGAGATACACAAGCAAACGACCAACAAAG
GAAGGAGCAAA

Coding Information

Region	Alignment Coordinates	Actual Coordinates
Exon 1 (position 1) GA24156 comp	1.. 5	16605742..16605746
Noncoding	6.. 116	16605747..16605839
Exon 1 (position 1) GA25052	117..434	16605840..16606172

This is the gene annotation in FlyBase for *D. pseudoobscura*. The pattern of nucleotide diversity is not consistent with a coding region. The dn/ds ratio for the partial sequence is fairly high and the outgroup *D. miranda* sequence has a partial deletion within the gene. The AUG start codon in gene GA25052 is polymorphic (AUG versus UUG). Therefore, we assume the following annotation.

>3:16605742,16606172
tttgctccttcctttgttggtcgtttgcttgtgtatctccaacatgggataccttccaca
ctcgctgggaaggacacagggcccctggtacttgttaacccgtctgtatccaccgatgca
gttgcaacggccatagcacaagacgtccacacaacgttggggataacaggacttggggca
gggattgccacaggctacctgcacctcgttcctgccgcagatggaactgccagagatagg
ggaatataggggaagatacagcccaaagatcagtgccagccggaacatggccaacgccaa
cgagtgccggagaagcattgtcgtgggagccctatcaaatggcttttgttctagctgtca
gagcgtggctaaaggtcttattcagttcagtttatacagttctctctccagttggtattt
caaaccatgaa

16 dHYSC, MV2-25

Ref Length= 361 bp, Aligned Length= 382 bp, Silent Sites w/o-Gaps= 323 bp

>3:17444491,17444852 (reverse complemented)
actagaattttggtgttgacggactgctgctcaacccttggactagttgtaaggcgacct
caaacatatcaagtagctattcttaatggagaaaggattcccaccaaaacactcttagat
cttcagctattgactttcgatgtgtactggtttttgttaccatattgtagactttagaat
cttcgtctgctcgatagaaacactaaggtagttgctattctaggtatactctgctttagg
tgtgtgccatatgttgttctgttgtaggtgcaacgaaagaaatggctaaaatcgatgggc
atttgtgagcaacacttaggaggcttcccttccgctggcagagtgtttctgttatgttca
gc

17 dHYST, MV2-25
Ref Length= 896 bp, Aligned Length= 923 bp, Silent Sites w/o-Gaps= 641 bp
```
>3:17705232,17706128
ttattcgaaagtttggattcgttcactcacAATTTCACCCAATAGGACAACATTTTCCCC
TCGGATTATAAACACTCCACGTGGAATGTCACCATATTGATTGCCCACATGTATCCTCTC
AATGGTGCGCTGCAAAACCAAGTTGGCAAACTGATCCACCGATCGTAGGTAGCCAATCAG
GGTCCGACCATCACGCAGGAGCACCATCAGTTTCTctgaaattaagacatttcattgtca
tccaaaaaaaacggccggagccactcacTGTCTACTTCTTCCAGGAGGTGAGCTGTGCCCG
CTAAAGGGTTCAGATCGTCCATgtgcagactgtgggtgattgattgtgtgtagaaaaacg
acttcgattcaacttttcgcttaattattgttaaatgtgtttttttttttcgagttctta
agtgctcctgctattcttgagtagcgtgggattatttggccgcccgactatgcgaagcag
gaaattgccaagtcaaaggatggaagttgttggtgtactagttataagtcaattagtgtt
cggcacacgctgatgttttctttttcaaaaaatttataaatatcattatactttcttaga
gatggcagtgcttcgataattcacgcgacagtgatcgcaggtggtggcagttcctcaata
gcacaatacaggaataaaatatcgatgcaaagtatcgattcaccgatatttttgattgtt
gacgttatcgagcagctgagccggcatgcggccaccattagtttgaaaaattgaaaattg
aatcgaaatagcaggaacATGAAgtaaatttgtgattaactgatctaaactgttgctaaa
accattggaaatccaacccttagGATCAGTATTGACGGCTTGCTCGTCTACTTCCCG
```

Coding Information (Note: This region includes two genes on opposite strands. GA25100 is on the plus strand and GA18077 is on the minus strand)
dHYST analysis of (324..923) GA25100 coding regions

Region	Alignment Coordinates	Actual Coordinates
Noncoding	1..819	17705232..17706030
Exon 1 (position 1) GA25100	820..824	17706031..17706035
Intron 1	825..889	17706036..17706095
Exon 2 (position 3) GA25100	890..923	17706096..17706129

dHYSTc analysis of (601..923) GA18077 coding regions

Region	Alignment Coordinates	Actual Coordinates
Noncoding	1..600	17706129..17705555
Exon 1 (position 1) GA18077	601..655	17705554..17705550
Intron 1	656..707	17705499..17705448
Exon 2 (position 2) GA18077	708..892	17705447..17705263
Intron 2	893..923	17705262..17705232

18 Ecdysone Receptor, MV2-25
Ref Length= 335 bp, Aligned Length= 343 bp, , Silent Sites w/o-Gaps= 127 bp
```
>3:17896399,17896734 (reverse complemented)
AGCGGCGCGAGAAGAAGGCCCAGAAGGAGAAGGACAAGATGACCACCTCGCCCAGCTCCC
AGCACGGCGGCAATGGCGCCGGGGGTGGTAATAGTGTGGGCGGCGGCTCCAGCCTTGGCG
GCGGCAGCGTCGGTGGTACCCATGACTACGTGAAGAAGGAGATTCTCGATCTGATGACAT
GCGAGGCGCCGCAGCATGCCACAATTCCGgtaagttgcagcaagcggatcgcaccagatt
ccctgactgatctcactctctctctctctcttctggcagCTACTACCTGATGATATTT
TGGCCAAGTGTCAAGCGCGCAATATACCTCCACTAA
```

Coding Information GA14596

Region	Alignment Coordinates	Actual Coordinates
Exon 2 (position 2)	1..209	17896734..
Intron 2	210..288	
Exon 3 (position 1)	289..343	..17896399

Frequency Spectra of Derived Mutations Within the Five Gene Arrangements. We used the *D. miranda* sequence to polarize the mutations within *D. pseudoobscura* to better understand the accumulation of new variants in the different gene arrangement backgrounds. We observed 758 polymorphic sites in the 18 regions. At 88.3 % of the 758 segregating sites, the most frequent *D. pseudoobscura* nucleotide matches the *D. miranda* nucleotide leading to the inference that the derived nucleotide is generally the lower frequency base. *D. miranda* has an inferred indel mutation at 3.3 % of the *D. pseudoobscura* segregating sites, which prevents an unambiguous inference of the derived base. For these sites, we made the conservative assumption that the majority base is the ancestral base, which will lead to an underestimate of the derived base frequency at the site if our ancestral base inference is wrong. Figure S3 shows the frequency spectra data for each segregating site.

Figure S4 shows the derived frequency spectra for segregating sites at breakpoint and non-breakpoint regions. The major difference between the two frequency spectra is in the relative frequencies of rare and fixed derived mutations. The breakpoint regions have a slightly lower frequency of rare derived variants and a higher frequency of fixed derived mutations than the non-breakpoint regions. In breakpoint regions, the frequency of rare variants increases as the age of the gene arrangement increases while this trend is less obvious in the non-breakpoint regions.

Unique, Shared and Fixed Polymorphisms Among Breakpoint and Non-breakpoint Regions of the *D. pseudoobscura* Gene Arrangements. We examined the distribution of the 758 polymorphisms among the five gene arrangements to determine whether they are unique to a particular arrangement, shared among arrangements, or represent a fixed difference within a gene arrangement. Each polymorphic site was classified into one of six categories based on the distribution of polymorphism among the five gene arrangements. Category 0 polymorphisms occurred when a segregating site was fixed in one to four gene arrangements. Category 1 polymorphisms occurred when a site was polymorphic in one arrangement and monomorphic in the other four arrangements (five possible configurations). Category 2 polymorphisms were defined when a site was polymorphic in two arrangements and monomorphic in the other three arrangements (ten possible combinations). Category 3 polymorphisms were indicated when a site was polymorphic in three arrangements and monomorphic in the other two arrangements (ten possible combinations). Category 4 polymorphisms occurred when a site was polymorphic in all, but one arrangement (five possible configurations). Finally, Category 5 polymorphisms were defined by sites that were polymorphic in all five arrangements.

The observed numbers of the six categories and 32 possible configurations of polymorphisms are shown in Table S7. We asked whether the observed frequencies of the polymorphism configurations depart from expectations derived from the observed polymorphism frequencies within the five arrangements. We used the fraction of sites that were polymorphic in each arrangement to estimate the joint probability for each outcome (AR, 302/ 758 = 0.398; PP 248/ 758 =0.327; ST, 218/ 758=

0.288; CH, 321 / 758=0.423; TL, 138/ 758= 0.182). If *p* is the fraction of polymorphic sites within arrangement, then the monomorphic fraction was obtained from 1- *p*. A chi-square goodness-of-fit test rejects the hypothesis that the distribution of polymorphisms among the five arrangements is independent (X^2=512.7, df=31, P=3.7 x 10^{-91}). The residuals show that there is an excess of category 1 polymorphisms (466 Observed versus 258.6 Expected), which are sites polymorphic in one arrangement and not in the others. The largest excess of sites occurs within the AR arrangement (140 Observed versus 68.3 Expected). Other configurations with a significant excess of sites are found in the Categories 3, 4, and 5. In category 3, there is a significant excess of sites where the polymorphism is shared among PP, CH, and TL. In category 4, AR, PP, ST, and CH are polymorphic and TL is monomorphic for more sites than expected (44 Observed versus 9.8 Expected). Of the 44 sites in this configuration, 11 sites represent fixations of the derived allele in the TL lineage.

We tested whether the distribution of polymorphic sites into the six categories was homogeneous between breakpoint and non-breakpoint regions with a chi-square test of homogeneity (Table S8). The category distribution is not homogeneous between breakpoint and non-breakpoint regions (X^2=25.4, df=5, P=0.0001). Two categories are responsible for rejecting the null hypothesis of homogeneity, categories 1 and 4 in non-breakpoint regions. Both cases are due to a significant deficiency of sites within non-breakpoint versus breakpoint loci.

We tested whether the frequency of unique polymorphisms within each arrangement was proportional to the frequency of total polymorphisms within each arrangement. We rejected the hypothesis of homogeneity with the AR arrangement having a significant excess of unique polymorphisms (X^2=16.5, *df*=4, *P*=0.002) (Table S9).

We observed 25 category 0 polymorphisms where all arrangements are monomorphic for a single nucleotide, but at least one arrangement is fixed for a derived variant. Of these polymorphisms, only one site is found in a non-breakpoint region (Amy1), six polymorphic sites in the dSTAR regions are fixed for the derived mutation only in the AR chromosome, and 11 polymorphic sites in the dHYSC region are fixed for the derived mutation in the TL arrangement. Overall, fixed derived mutations in just the TL chromosome accounts for 14 of the 25 category 0 polymorphic sites, which is greater than expected given the relative proportion of polymorphic sites in TL (X^2=30.5, df=2, P=3.4x10^{-7}) (Table S10).

Overall, this analysis shows that each arrangement has accumulated unique polymorphisms with the AR, one of the youngest arrangements, having the greatest number of unique polymorphisms (Table S7). Breakpoint regions, however, do not have a significant excess of unique polymorphisms compared to non-breakpoint regions (Table S8).
