# Supplementary material for: Molecular Population Genetics of Inversion Breakpoint Regions in Drosophila pseudoobscura
Source: G3 (Bethesda). 2013 Jul 1;3(7):1151–63. doi: 10.1534/g3.113.006122 (PMC3704243; doi:10.1534/g3.113.006122)
Supplement: Supporting Information [file supp_g3.113.006122_TableS1.pdf]

Table S1 Primer Sequences Used for PCR Amplification

Primer Name	Primer Sequence	Coordinate Interval	Length (bp)	Cytological Position	Annealing Temp (° C)
pSTPP_f	GAT ACC ACT CGG CAA GCA GAA G	2,292,752…2,293,105	354	64C	55
pSTPP_r	CGC CTC AGT TAA TTA GCC CAC AAA				
pHYSC_f	TGG TGT TGA GTA TCT GCC GTG GTT	6,432,216…6,432,591	376	68C	60
pHYSC_r	CTG CTG CCG CTG CTC CTA TCA				
pSTAR_f	CCT GAT ACC CAC GGA GTC TTC	8,900,356…8,900,823	468	76B	55
pSTAR_r	TCG CTA CAG GGA TCA GGT TTT				
pHYST_f	CTT ATT CCC GCC TCT TGT GTA GC	9,140,888..9,141,693	806	76B	60
pHYST_r	GAC GGC CCT CAG ACG ATA GTT G				
dSTPP_f	ATC GGT ACA ACA GCC AGG GAC AAC	9,832,256…9,832,828	573	75B	58
dSTPP_r	ACT TCG TGG GAT CGC TGG CAT AAT				
dSCTL_f	ATG GCG ATG GAG TCC TCT GTC TAT	10,830,478...10,830,881	404	74B	60
dSCTL_r	ACT GGC GCC ATG TCT CTG TCT CG				
pSCCH_f	AAC CGG CAT ACA CCC TCA TTC	14,259,606…14,259,984	377	70C	57
pSCCH_r	GTT GCG CAT TAT TTA TTC CCT GTA				
dSCCH_f	TCC GGA GAT CGC AAA ACT GTC G	15426293…15426646	379	77B	55
dSCCH_r	TAT GCG CTG CTT CTG ATG CTT GAT				
dHYSC_f	GAG CCC GGG CCA GGT CCA T	17,444,491…17,444,852	362	79C	58
dHYSC_r	TAT CGT GCG TTG TGC GTA ATC AGC				

dHYST_f	ACA AGA TCC GGG GTA TTA	17,705,2323..17,706,128	898	79D/80A
dHYST_r	CTG TTC CGG GTA GAT GTA TTC GTA			

The abbreviated name represents the location (p, proximal or d, distal) of the breakpoint on the chromosome, and the last four letters represent the two arrangements involved in the inversion. The first two letters are the ancestral arrangement and the last two letters are the derived arrangement. For example, the STPP notation is for the breakpoints that converted the ancestral Standard arrangement into the derived Pikes Peak arrangement. Primer names are the abbreviated region names with the addition of “f” and “r” for forward or reverse. The coordinates are the location of the genetic marker in the genome strain (Richards *et al.* 2005), which carries the Arrowhead arrangement.
