# Supplementary material for: Molecular Population Genetics of Inversion Breakpoint Regions in Drosophila pseudoobscura
Source: G3 (Bethesda). 2013 Jul 1;3(7):1151–63. doi: 10.1534/g3.113.006122 (PMC3704243; doi:10.1534/g3.113.006122)
Supplement: Supporting Information [file supp_g3.113.006122_TableS2.pdf]

Table S2 Nucleotide Polymorphism in 18 Regions of the *D. pseudoobscura* Third Chromosome.

Region **(Silent Sites)**	***Sample***	***n***	***S***	***θ ± Sd***	***k***	***π ± Sd***	***Tajima's D***
**pSTPP (353 bp)**	All	91	26	0.014 ± 0.004	3.519	0.010 ± 0.005	-0.945
	AR	23	14	0.011 ± 0.004	2.842	0.008 ± 0.004	-0.887
	PP	20	12	0.010 ± 0.004	3.321	0.009 ± 0.005	-0.065
	ST	21	11	0.009 ± 0.004	2.919	0.008 ± 0.005	-0.159
	CH	22	11	0.009 ± 0.004	3.108	0.009 ± 0.005	0.104
	TL	4	2	0.003 ± 0.002	1.000	0.003 ± 0.002	-0.710*
en **(302 bp)**	All	142	46	0.029 ± 0.008	3.603	0.012 ± 0.006	-1.728*
	AR	50	27	0.020 ± 0.007	2.830	0.009 ± 0.005	-1.748*
	PP	25	8	0.007 ± 0.003	1.740	0.006 ± 0.003	-0.567
	ST	27	18	0.015 ± 0.006	2.621	0.009 ± 0.005	-1.544
	CH	32	15	0.012 ± 0.005	4.763	0.016 ± 0.008	0.927
	TL	7	9	0.012 ± 0.007	3.428	0.011 ± 0.007	-0.355
**pHYSC/pSCTL (193 bp)**	All	93	27	0.027 ± 0.008	5.200	0.027 ± 0.013	-0.051
	AR	23	6	0.008 ± 0.004	0.885	0.005 ± 0.003	-1.394
	PP	19	15	0.022 ± 0.009	4.971	0.026 ± 0.013	0.592
	ST	23	11	0.015 ± 0.007	1.375	0.007 ± 0.005	-1.843*
	CH	23	12	0.017 ± 0.007	2.300	0.012 ± 0.007	-1.014
	TL	4	11	0.031 ± 0.018	5.500	0.028 ± 0.017	-0.837
exu 1 **(176 bp)**	All	144	17	0.018 ± 0.006	1.312	0.007 ± 0.005	-1.546
	AR	53	8	0.010 ± 0.004	1.066	0.006 ± 0.004	-1.067
	PP	26	3	0.004 ± 0.003	0.671	0.004 ± 0.003	-0.359
	ST	25	5	0.008 ± 0.004	1.319	0.008 ± 0.005	-0.011

	CH	32	5	0.007 ± 0.004	1.402	0.008 ± 0.005	0.347
	TL	7	2	0.005 ± 0.004	0.762	0.004 ± 0.004	-0.274
**pSTAR (440 bp)**	All	90	83	0.037 ± 0.010	9.559	0.022 ± 0.010	-1.374
	AR	24	35	0.021 ± 0.008	8.221	0.019 ± 0.009	-0.468
	PP	19	31	0.020 ± 0.008	8.795	0.020 ± 0.010	-0.033
	ST	22	45	0.028 ± 0.010	9.918	0.023 ± 0.011	-0.772
	CH	20	41	0.026 ± 0.010	10.147	0.023 ± 0.011	-0.487
	TL	4	10	0.012 ± 0.007	5.167	0.012 ± 0.007	-0.527
**pHYST 484 bp)**	All	90	59	0.024 ± 0.007	8.854	0.018 ± 0.009	-0.776
	AR	23	29	0.016 ± 0.006	7.154	0.015 ± 0.007	-0.340
	PP	19	33	0.019 ± 0.007	10.443	0.022 ± 0.010	0.423
	ST	19	10	0.006 ± 0.003	3.846	0.008 ± 0.004	1.221
	CH	24	23	0.013 ± 0.005	5.594	0.012 ± 0.006	-0.339
	TL	4	21	0.024 ± 0.013	10.501	0.022 ± 0.013	-0.854
**dSTPP (523 bp)**	All	90	43	0.016 ± 0.005	7.918	0.015 ± 0.007	-0.210
	AR	24	21	0.011 ± 0.004	3.896	0.007 ± 0.004	-1.125
	PP	19	4	0.002 ± 0.001	0.926	0.002 ± 0.001	-0.562
	ST	19	7	0.004 ± 0.002	1.109	0.002 ± 0.001	-1.490
	CH	22	22	0.012 ± 0.004	5.104	0.010 ± 0.005	-0.578
	TL	4	0	0.000 ± 0.000	0.000	0.000 ± 0.000	
**dSCTL (374 bp)**	All	92	47	0.025 ± 0.007	8.242	0.022 ± 0.010	-0.341
	AR	22	11	0.008 ± 0.004	2.649	0.007 ± 0.004	-0.423
	PP	19	17	0.013 ± 0.005	4.749	0.013 ± 0.006	-0.090
	ST	22	9	0.007 ± 0.003	2.009	0.005 ± 0.003	-0.624
	CH	23	22	0.016 ± 0.006	4.893	0.013 ± 0.007	-0.665

	TL	4	8	0.012 ± 0.007	5.000	0.013 ± 0.008	1.442
***eve* (146 bp)**	All	142	24	0.031 ± 0.010	3.337	0.023 ± 0.012	-0.659
	AR	51	6	0.009 ± 0.004	1.471	0.010 ± 0.006	0.261
	PP	26	10	0.018 ± 0.008	3.441	0.024 ± 0.012	1.025
	ST	26	2	0.004 ± 0.003	0.224	0.002 ± 0.002	-1.226
	CH	31	17	0.029 ± 0.011	3.015	0.021 ± 0.011	-0.991
	TL	7	0	0.000 ± 0.000	0.000	0.000 ± 0.000	
***Mef 2* (315 bp)**	All	125	43	0.026 ± 0.007	5.122	0.016 ± 0.008	-1.095
	AR	40	10	0.007 ± 0.003	1.654	0.005 ± 0.003	-0.884
	PP	26	12	0.010 ± 0.004	2.718	0.009 ± 0.005	-0.457
	ST	24	10	0.009 ± 0.004	1.166	0.004 ± 0.002	-1.884*
	CH	27	17	0.014 ± 0.005	3.534	0.011 ± 0.006	-0.695
	TL	7	12	0.016 ± 0.008	3.808	0.012 ± 0.007	-1.212
***Amy 1* (363 bp)**	All	142	51	0.027 ± 0.007	5.369	0.015 ± 0.007	-1.284
	AR	50	20	0.012 ± 0.004	3.082	0.008 ± 0.004	-0.988
	PP	25	3	0.002 ± 0.001	0.661	0.002 ± 0.001	-0.417
	ST	27	13	0.009 ± 0.004	2.323	0.006 ± 0.004	-1.051
	CH	32	11	0.008 ± 0.003	1.942	0.005 ± 0.003	-0.917
	TL	7	13	0.015 ± 0.007	5.046	0.014 ± 0.008	-0.268
**pSCCH (326 bp)**	All	86	29	0.018 ± 0.005	5.026	0.015 ± 0.008	-0.398
	AR	21	7	0.006 ± 0.003	0.981	0.003 ± 0.002	-1.607
	PP	20	8	0.007 ± 0.003	3.747	0.011 ± 0.006	2.230*
	ST	21	5	0.004 ± 0.002	0.476	0.001 ± 0.001	-1.982*
	CH	19	8	0.007 ± 0.003	3.275	0.010 ± 0.005	1.474
	TL	4	0	0.000 ± 0.000	0.000	0.000 ± 0.000	

**dSTAR(*vg*) (309 bp)**	All	143	95	0.058 ± 0.015	17.799	0.058 ± 0.026	0.119
	AR	52	20	0.014 ± 0.005	3.319	0.011 ± 0.006	-0.793
	PP	23	27	0.024 ± 0.009	7.858	0.025 ± 0.012	0.280
	ST	28	25	0.021 ± 0.008	7.818	0.025 ± 0.012	0.782
	CH	31	43	0.035 ± 0.012	5.040	0.016 ± 0.008	-1.956*
	TL	7	33	0.044 ± 0.021	11.097	0.036 ± 0.019	-1.008
**dSCCH (85 bp)**	All	90	8	0.019 ± 0.008	1.417	0.017 ± 0.010	-0.251
	AR	22	4	0.013 ± 0.007	1.389	0.016 ± 0.010	0.747
	PP	20	1	0.003 ± 0.003	0.101	0.001 ± 0.002	-1.158
	ST	22	1	0.003 ± 0.003	0.369	0.004 ± 0.004	0.600
	CH	21	2	0.007 ± 0.005	0.560	0.007 ± 0.006	0.017
	TL	4	2	0.013 ± 0.010	1.338	0.016 ± 0.012	1.929*
**F6 (428 bp)**	All	126	57	0.025 ± 0.007	6.463	0.015 ± 0.007	-1.210
	AR	45	31	0.017 ± 0.005	3.663	0.009 ± 0.004	-1.638
	PP	24	24	0.015 ± 0.006	4.598	0.011 ± 0.005	-1.055
	ST	25	16	0.010 ± 0.004	3.367	0.008 ± 0.004	-0.725
	CH	24	22	0.014 ± 0.005	4.533	0.011 ± 0.005	-0.848
	TL	7	5	0.005 ± 0.003	2.571	0.006 ± 0.004	1.287
**dHYSC (323 bp)**	All	87	39	0.024 ± 0.007	4.421	0.014 ± 0.007	-1.359
	AR	24	11	0.009 ± 0.004	3.094	0.010 ± 0.005	0.171
	PP	19	11	0.010 ± 0.004	2.795	0.009 ± 0.005	-0.402
	ST	21	10	0.009 ± 0.004	3.248	0.010 ± 0.005	0.582
	CH	18	12	0.011 ± 0.005	2.497	0.008 ± 0.004	-1.050
	TL	4	7	0.012 ± 0.007	3.500	0.011 ± 0.007	-0.817

**dHYST (641 bp)**	All	94	54	0.016 ± 0.005	8.600	0.013 ± 0.006	-0.596
	AR	24	27	0.011 ± 0.004	9.115	0.014 ± 0.007	0.975
	PP	19	30	0.013 ± 0.005	10.256	0.016 ± 0.008	0.773
	ST	23	18	0.008 ± 0.003	4.593	0.007 ± 0.004	-0.212
	CH	23	36	0.015 ± 0.005	7.125	0.011 ± 0.005	-1.037
	TL	4	0	0.000 ± 0.000	0.000	0.000 ± 0.000	
**EcR (127 bp)**	All	142	21	0.031 ± 0.010	1.532	0.012 ± 0.007	-1.669*
	AR	51	16	0.028 ± 0.010	1.793	0.014 ± 0.008	-1.530
	PP	25	6	0.012 ± 0.006	1.073	0.008 ± 0.006	-0971
	ST	26	4	0.008 ± 0.005	1.323	0.010 ± 0.007	0.700
	CH	32	8	0.016 ± 0.007	1.573	0.012 ± 0.008	-0.623
	TL	7	2	0.006 ± 0.005	0.572	0.004 ± 0.004	-1.234

n, sample size; *S,* number of segregating sites; $\theta \pm$ sd, nucleotide heterozygosity per site based on the number of segregating sites with its standard deviation (WATTERSON 1975); $\pi \pm$ sd, nucleotide heterozygosity per site based on the number of pairwise differences with its standard deviation assuming no recombination (TAJIMA 1983); Tajima's (1989) *D*, a test of the frequency spectrum's departure from expectations of the neutral theory where values of Tajima's *D* marked with an asterisk have a probability less than 0.05.
