# Supplementary material for: Molecular Population Genetics of Inversion Breakpoint Regions in Drosophila pseudoobscura
Source: G3 (Bethesda). 2013 Jul 1;3(7):1151–63. doi: 10.1534/g3.113.006122 (PMC3704243; doi:10.1534/g3.113.006122)
Supplement: Supporting Information [file supp_g3.113.006122_TableS3.pdf]

Table S3 HKA test for the Arrowhead gene arrangement

Gene	AR_Obs_S	AR_Exp_S	Dmir_Obs_D	AR_Exp_D
pSTPP	14	11.04	5.28	8.25
en	27	18.74	3.28	11.54
pHYSC	6	8.40	8.68	6.28
exu1	8	5.57	0.96	3.39
pSTAR	35	33.29	22.87	24.59
pHYST	29	28.64	21.03	21.40
dSTPP	21	17.70	9.77	13.07
dSCTL	11	14.10	13.77	10.67
eve	6	6.56	4.58	4.02
Mef2	10	13.42	12.12	8.70
Amy1	20	17.74	8.66	10.92
pSCCH	7	11.72	13.71	8.99
dSTAR	20	24.39	19.28	14.89
dSCCH	4	4.42	3.77	3.35
F6	31	26.80	12.70	16.90
dHYSC	11	12.93	11.48	9.55
dHYST	27	35.41	34.56	26.15
EcR	16	12.13	3.57	7.44
T	1.76			
X2	16.17			
P	0.212			
sim	9982			
