# Supplementary material for: Molecular Population Genetics of Inversion Breakpoint Regions in Drosophila pseudoobscura
Source: G3 (Bethesda). 2013 Jul 1;3(7):1151–63. doi: 10.1534/g3.113.006122 (PMC3704243; doi:10.1534/g3.113.006122)
Supplement: Supporting Information [file supp_g3.113.006122_FileS2.pdf]

File S2

Literature Cited

RICHARDS, S., Y. LIU, B. R. BETTENCOURT, P. HRADECKY, S. LETOVSKY *et al.*, 2005 Comparative genome sequencing of *Drosophila pseudoobscura*: Chromosomal, gene and *cis*-element evolution. Genome Research **15:** 1-18.

TAJIMA, F., 1983 Evolutionary relationship of DNA sequences in finite populations. Genetics **105:** 437-460.

TAJIMA, F., 1989 Statistical method for testing the neutral mutation hypothesis by DNA polymorphism. Genetics **123:** 585-595.

WATTERSON, G. A., 1975 On the number of segregating sites in genetical models without recombination. Theoretical Population Biology **7:** 256-276.
