# Supplementary material for: Molecular Population Genetics of Inversion Breakpoint Regions in Drosophila pseudoobscura
Source: G3 (Bethesda). 2013 Jul 1;3(7):1151–63. doi: 10.1534/g3.113.006122 (PMC3704243; doi:10.1534/g3.113.006122)
Supplement: Supporting Information [file supp_g3.113.006122_TableS4.pdf]

Table S4 HKA test for the Pikes Peak gene arrangement

Gene	PP_Obs_S	PP_Exp_S	Dmir_Obs_D	PP_Exp_D
pSTPP	12	9.43	5.28	7.85
en	8	6.33	3.28	4.95
pHYSC	15	12.83	8.68	10.84
exu1	3	2.23	0.96	1.73
pSTAR	31	29.2	22.87	24.68
pHYST	33	29.29	21.03	24.75
dSTPP	4	7.46	9.77	6.31
dSCTL	17	16.68	13.77	14.09
eve	10	8.22	4.58	6.36
Mef2	12	13.6	12.12	10.52
Amy1	3	6.54	8.66	5.12
pSCCH	8	11.84	13.71	9.86
dSTAR	27	25.71	19.28	20.57
dSCCH	1	2.6	3.77	2.17
F6	24	20.49	12.70	16.21
dHYSC	11	12.18	11.48	10.3
dHYST	30	34.99	34.56	29.57
EcR	6	5.37	3.57	4.2
T	1.95			
X2	11.67			
P	0.559			
sim	9639			
