# Supplementary material for: Molecular Population Genetics of Inversion Breakpoint Regions in Drosophila pseudoobscura
Source: G3 (Bethesda). 2013 Jul 1;3(7):1151–63. doi: 10.1534/g3.113.006122 (PMC3704243; doi:10.1534/g3.113.006122)
Supplement: Supporting Information [file supp_g3.113.006122_TableS5.pdf]

**Table S5 HKA test for the Standard gene arrangement**

| Gene | ST_Obs_S | ST_Exp_S | Dmir_Obs_D | ST_Exp_D |
|---|---|---|---|---|
| pSTPP | 11 | 8.22 | 5.28 | 8.07 |
| en | 18 | 11.11 | 3.28 | 10.18 |
| pHYSC | 11 | 10.05 | 8.68 | 9.62 |
| exu1 | 5 | 3.08 | 0.96 | 2.88 |
| pSTAR | 45 | 34.47 | 22.87 | 33.4 |
| pHYST | 10 | 15.44 | 21.03 | 15.6 |
| dSTPP | 7 | 8.34 | 9.77 | 8.43 |
| dSCTL | 9 | 11.56 | 13.77 | 11.2 |
| eve | 2 | 3.41 | 4.58 | 3.16 |
| Mef2 | 10 | 11.37 | 12.12 | 10.75 |
| Amy1 | 13 | 11.3 | 8.66 | 10.36 |
| pSCCH | 5 | 9.44 | 13.71 | 9.27 |
| dSTAR | 25 | 23.31 | 19.28 | 20.97 |
| dSCCH | 1 | 2.42 | 3.77 | 2.35 |
| F6 | 16 | 14.83 | 12.70 | 13.87 |
| dHYSC | 10 | 10.84 | 11.48 | 10.64 |
| dHYST | 18 | 26.86 | 34.56 | 25.7 |
| EcR | 4 | 3.93 | 3.57 | 3.64 |
| T | 2.53 | | | |
| X2 | 18.22 | | | |
| P | 0.145 | | | |
| sim | 9813 | | | |
