# Supplementary material for: Molecular Population Genetics of Inversion Breakpoint Regions in Drosophila pseudoobscura
Source: G3 (Bethesda). 2013 Jul 1;3(7):1151–63. doi: 10.1534/g3.113.006122 (PMC3704243; doi:10.1534/g3.113.006122)
Supplement: Supporting Information [file supp_g3.113.006122_TableS6.pdf]

Table S6   HKA test for the Chiracahua gene arrangement

Gene	CH_Obs_S	CH_Exp_S	Dmir_Obs_D	CH_Exp_D
pSTPP	11	9.81	5.28	6.47
en	15	11.45	3.28	6.83
pHYSC	12	12.52	8.68	8.16
exu1	5	3.73	0.96	2.23
pSTAR	41	38.07	22.87	25.8
pHYST	23	26.79	21.03	17.25
dSTPP	22	19.14	9.77	12.63
dSCTL	22	21.66	13.77	14.11
eve	17	13.47	4.58	8.11
Mef2	17	17.93	12.12	11.19
Amy1	11	12.31	8.66	7.35
pSCCH	8	12.86	13.71	8.85
dSTAR	43	38.88	19.28	23.4
dSCCH	2	3.46	3.77	2.31
F6	22	21.11	12.70	13.59
dHYSC	12	13.82	11.48	9.66
dHYST	36	42.73	34.56	27.83
EcR	8	7.25	3.57	4.33
T	1.4			
X2	8.28			
P	0.783			
sim	9875			
