# Supplementary material for: Molecular Population Genetics of Inversion Breakpoint Regions in Drosophila pseudoobscura
Source: G3 (Bethesda). 2013 Jul 1;3(7):1151–63. doi: 10.1534/g3.113.006122 (PMC3704243; doi:10.1534/g3.113.006122)
Supplement: Supporting Information [file supp_g3.113.006122_TableS7.pdf]

Table S7 Shared, unique, and fixed polymorphisms among the five gene arrangements of *D. pseudoobscura*.

Category	Configuration	Obs	Exp	Residuals
0	P() M(AR, PP, ST, CH, TL)	25	103.1	-7.7
1	P(AR) M(PP, ST, CH, TL)	140	68.3	8.7
1	P(PP) M(AR, ST, CH, TL)	81	50.1	4.4
1	P(ST) M(AR, PP, CH, TL)	68	41.6	4.1
1	P(CH) M(AR, PP, ST, TL)	126	75.7	5.8
1	P(TL) M(AR, PP, ST, CH)	51	22.9	5.9
2	P(AR, PP) M(ST, CH, TL)	14	33.2	-3.3
2	P(AR, ST) M(PP, CH, TL)	12	27.6	-3.0
2	P(AR, CH) M(PP, ST, TL)	30	50.1	-2.8
2	P(AR, TL) M(PP, ST, CH)	4	15.2	-2.9
2	P(PP, ST) M(AR, CH, TL)	5	20.2	-3.4
2	P(PP, CH) M(AR, ST, TL)	13	36.8	-3.9
2	P(PP, TL) M(AR, ST, CH)	10	11.2	-0.3
2	P(ST, CH) M(AR, PP, TL)	23	30.6	-1.4
2	P(ST, TL) M(AR, PP, CH)	5	9.3	-1.4
2	P(CH, TL) M(AR, PP, ST)	6	16.9	-2.6
3	P(AR, PP, ST) M(CH, TL)	13	13.4	-0.1
3	P(AR, PP, CH) M(ST, TL)	11	24.4	-2.7
3	P(AR, PP, TL) M(ST, CH)	5	7.4	-0.9
3	P(AR, ST, CH) M(PP, TL)	8	20.2	-2.7
3	P(AR, ST, TL) M(PP, CH)	1	6.1	-2.1
3	P(AR, CH, TL) M(PP, ST)	2	11.2	-2.7
3	P(PP, ST, CH) M(AR, TL)	7	14.9	-2.0
3	P(PP, ST, TL) M(AR, CH)	3	4.5	-0.7
3	P(PP, CH, TL) M(AR, ST)	16	8.2	2.7
3	P(ST, CH, TL) M(AR, PP)	6	6.8	-0.3
4	P(AR, PP, ST, CH) M(TL)	44	9.8	10.9
4	P(AR, PP, ST, TL) M(CH)	0	3.0	-1.7
4	P(AR, PP, CH, TL) M(ST)	6	5.4	0.2
4	P(AR, ST, CH, TL) M(PP)	3	4.5	-0.7
4	P(PP, ST, CH, TL) M(AR)	11	3.3	4.2
5	P(AR, PP, ST, CH, TL) M()	9	2.2	4.6

Category, polymorphism category; Configuration, P() and M() indicate which arrangements were polymorphic or monomorphic at a site, respectively. Obs, observed numbers of each configuration; Exp, expected number of each configuration assuming that the polymorphisms are distributed independently. The colored boxes in the residuals column indicate deficiencies (blue) or excesses (red) of polymorphic sites for a particular configuration.
