# Supplementary material for: Molecular Population Genetics of Inversion Breakpoint Regions in Drosophila pseudoobscura
Source: G3 (Bethesda). 2013 Jul 1;3(7):1151–63. doi: 10.1534/g3.113.006122 (PMC3704243; doi:10.1534/g3.113.006122)
Supplement: Supporting Information [file supp_g3.113.006122_TableS8.pdf]

Table S8 Observed and expected numbers of polymorphic sites classified into the six unique, shared, and fixed categories for the five gene arrangements of *D. pseudoobscura*.

Category	Breakpoint	Non-Breakpoint
0	24 (16.8)	1 (8.2)
1	298 (312.9)	168 (153.1)
2	80 (81.9)	42 (40.1)
3	44 (48.3)	28 (23.7)
4	56 (43.0)	8 (21.0)
5	7 (6.0)	2 (3.0)

Observed (Expected); The blue shaded box indicates a significant deficiency of observed values based on the residuals.
