# Supplementary material for: Molecular Population Genetics of Inversion Breakpoint Regions in Drosophila pseudoobscura
Source: G3 (Bethesda). 2013 Jul 1;3(7):1151–63. doi: 10.1534/g3.113.006122 (PMC3704243; doi:10.1534/g3.113.006122)
Supplement: Supporting Information [file supp_g3.113.006122_TableS9.pdf]

Table S9 Observed and (expected) numbers of unique polymorphic sites for the five gene arrangements of *D. pseudoobscura*.

Arrangement	Unique Polymorphisms	Non-Unique Polymorphisms
CH	126 (121.9)	195 (199.1)
AR	140 (114.7)	162 (187.3)
PP	81 (153.8)	167 (153.8)
ST	68 (82.8)	150 (135.2)
TL	51 (52.4)	87 (85.6)
