# Supplementary material for: Molecular Population Genetics of Inversion Breakpoint Regions in Drosophila pseudoobscura
Source: G3 (Bethesda). 2013 Jul 1;3(7):1151–63. doi: 10.1534/g3.113.006122 (PMC3704243; doi:10.1534/g3.113.006122)
Supplement: Supporting Information [file supp_g3.113.006122_TableS10.pdf]

Table S10 Observed and (expected) numbers of category 0 polymorphic sites for the three gene arrangements of *D. pseudoobscura*.

Arrangement	Unique Fixed Polymorphisms	Non-Unique Fixed Polymorphisms
AR	6 (9.2)	296 (292.8)
PP	1 (7.6)	247 (240.4)
TL	14 (4.2)	124 (133.8)
